# Coupled Transport, Plasticization, and Retention Mechanisms in Phosphoric Acid-Doped PBI Membranes

**DOI:** 10.3390/membranes16060210

**Published:** 2026-06-17

**Authors:** Francesca Stella, Sergio Bocchini

**Affiliations:** 1 Center for Sustainable Future Technologies (CSFT), Istituto Italiano di Tecnologia (IIT), Via Livorno 60, 10144 Turin, Italy; francesca.stella@iit.it; 2 Department of Applied Science and Technology, Politecnico di Torino, Corso Duca Degli Abruzzi 24, 10129 Turin, Italy

**Keywords:** PA–PBI membranes, high-temperature PEM fuel cells, proton conductivity, acid doping level, chemo-mechanical coupling, acid retention, membrane durability, hydrogen-bond networks, plasticization, multiscale design

## Abstract

Phosphoric acid-doped polybenzimidazole membranes are a leading fluorine-free electrolyte platform for high-temperature proton exchange membrane fuel cells, enabling proton transport under anhydrous conditions. However, recent evidence shows that conductivity, mechanical stability, and acid retention are intrinsically coupled, preventing independent optimization of these properties. This review establishes a unified framework in which membrane performance is governed by a multidimensional design space defined by acid doping level, activation energy (E_a_), hydrogen-bond network topology, and mechanical confinement. Conductivity is shown to scale with both carrier density and hopping energetics, while mechanical stability decays with increasing ADL due to acid-induced plasticization, described through a semi-empirical relationship. Analysis across molecular architectures, including molecular weight control, crosslinking, backbone modification, topological design, and free-volume engineering, demonstrates that performance emerges from a balance between transport efficiency and structural stability. Device-level benchmarking further reveals that similar conductivity values can correspond to orders-of-magnitude differences in voltage decay rate, confirming that durability is governed primarily by mechanical confinement and acid mobility rather than σ alone. A multivariate stability corridor is identified, within which phosphoric acid-doped polybenzimidazole membranes achieve σ ≈ 0.14–0.20 S·cm^−1^ while maintaining low degradation rates under realistic high temperature proton exchange membrane conditions. Based on this framework, quantitative design rules are derived linking acid doping level, activation, topology, and mechanical properties. This work shifts membrane design from conductivity-driven optimization toward predictive structure–property–durability engineering, providing a basis for the development of next-generation HT-PEM fuel cells with sustained long-term performance.

## 1. Introduction

### 1.1. From Hydrated Proton Conduction to High-Temperature Polymer Electrolytes

Proton exchange membrane fuel cells (PEMFCs) are among the most mature electrochemical technologies for hydrogen-based energy conversion. Their high-power density, rapid start-up, and compatibility with renewable hydrogen have positioned them as key components in decarbonization strategies for transportation, stationary power, and distributed energy systems. Over the past three decades, PEMFC development has been largely driven by low-temperature systems (<100 °C) employing perfluorosulfonic acid (PFSA) membranes.

**Figure 1:**
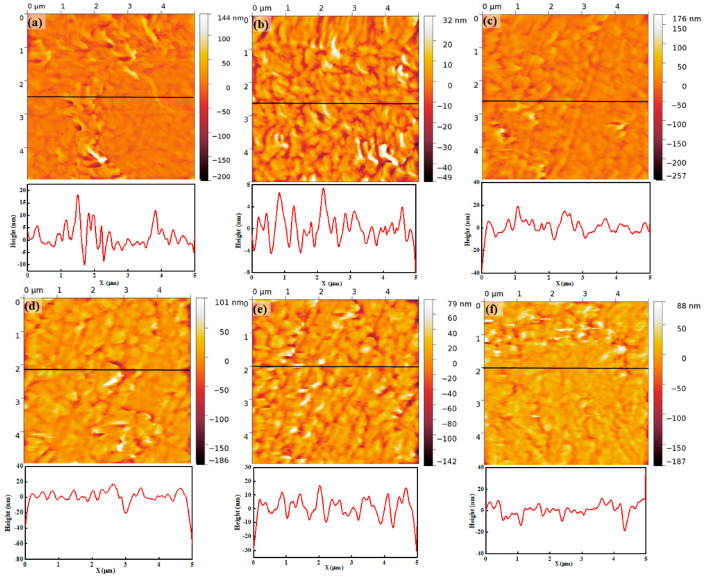
Schematic illustration of the phase-separated morphology and ionic channel network in Nafion. Hydrophilic domains associated with sulfonic acid groups form interconnected water-filled channels that enable proton transport. The degree of channel connectivity and percolation increases with hydration, providing a model system for understanding proton conduction mechanisms in polymer electrolyte membranes. Topography and related line profiles of Nafion 115 under RH values of (**a**) 15%, (**b**) 25%, (**c**) 45%, (**d**) 55%, (**e**) 65%, and (**f**) 75%. Adapted from Figure 6 Ref. [1].

In PFSA membranes, proton transport is governed by phase-separated hydrated ionic domains formed by sulfonic acid groups tethered to a fluorinated backbone. This morphology consists of nanoscopic hydrophilic clusters embedded in a hydrophobic matrix, enabling the formation of percolated water channels. As hydration increases, these domains expand and interconnect, facilitating efficient proton transport. Conversely, at low relative humidity, domain contraction disrupts percolation pathways, resulting in a sharp decline in conductivity (Figure 1).

Extensive studies have shown that conductivity in PFSA and related systems strongly depends on water uptake, microphase separation, and equivalent weight [2,3,4,5]. Under fully hydrated conditions, well-developed water channels enable proton transport via vehicular and Grotthuss mechanisms, with conductivity exceeding 0.1 S·cm^−1^. However, this performance is intrinsically linked to hydration. As water content decreases, ionic domains contract, transport pathways fragment, and conductivity drops rapidly.

This hydration dependence imposes significant operational constraints. Continuous humidification is required to maintain ionic conductivity and ensure the formation of percolated proton-conducting pathways. However, excessive water accumulation is equally detrimental. At high current densities, liquid water can accumulate within the gas diffusion layer and catalyst layer, leading to flooding phenomena that obstruct gas transport and reduce reactant accessibility. This results in increased mass transport losses and rapid performance degradation. Therefore, PEMFC operation requires a delicate balance between membrane hydration and effective water removal. Non-uniform hydration can further induce mechanical stress, membrane thinning, and local degradation, necessitating tight control of operating conditions and optimized water management strategies [6,7,8,9].

Additional limitations arise from low-temperature operation. Platinum-based anodes exhibit high sensitivity to CO adsorption below 100 °C, restricting the use of reformate hydrogen without extensive purification. Furthermore, low operating temperatures limit thermal integration with upstream reformers and downstream heat recovery systems, reducing overall system efficiency.

Environmental considerations have further accelerated the search for alternatives to PFSA membranes. The persistence of perfluorinated compounds has raised concerns regarding their long-term environmental impact, motivating the development of fluorine-free polymer electrolytes. While hydrocarbon membranes offer potential sustainability advantages, many suffer from limited chemical stability or insufficient conductivity under comparable conditions [10,11,12].

These limitations have driven the development of high-temperature PEM fuel cells (HT-PEMFCs), operating in the 120–200 °C range under low or zero external humidification. Elevated temperatures improve electrode kinetics, enhance CO tolerance, and simplify thermal management, enabling more efficient system integration.

However, operation under anhydrous high-temperature conditions requires proton transport mechanisms that do not rely on bulk water. The transport paradigm must therefore shift from hydration-mediated percolation to alternative hydrogen-bonded or acid-based networks capable of sustaining conductivity in dry environments. This transition defines the fundamental challenge of HT-PEM electrolyte design.

Among hydrocarbon-based materials, phosphoric acid-doped polybenzimidazole (PA–PBI) has emerged as a leading candidate [13,14,15]. In contrast to PFSA membranes, where conductivity is governed by hydrated ionic domains, PA–PBI systems rely on acid–base interactions between benzimidazole groups and phosphoric acid. Proton transport occurs through hydrogen-bonded phosphoric acid networks stabilized within the polymer matrix, enabling significant conductivity under anhydrous conditions [16,17].

This transition from hydration-controlled to acid-network-mediated transport fundamentally redefines membrane design variables. While PFSA systems are governed by water uptake and microphase morphology, PA–PBI membranes are controlled by acid doping level (ADL), proton transfer activation energy, and structural confinement of the polymer backbone. Thus, the shift to high-temperature operation represents not only a change in operating conditions, but a transformation in the underlying transport mechanism and design philosophy.

This hydration dependence imposes significant operational constraints. Continuous humidification is required to maintain performance, increasing system complexity and cost. Non-uniform hydration can also induce mechanical stress, membrane thinning, and local degradation, necessitating tight control of operating conditions [5,6,7].

These limitations have driven the development of HT-PEMFCs, operating in the 120–200 °C range under low or zero external humidification. Elevated temperatures improve electrode kinetics, enhance CO tolerance, and simplify thermal management, enabling more efficient system integration.

Among hydrocarbon-based materials, phosphoric PA–PBI has emerged as a leading candidate [13,14,15]. In contrast to PFSA membranes, where conductivity is governed by hydrated ionic domains, PA–PBI systems rely on acid–base interactions between benzimidazole groups and phosphoric acid. Proton transport occurs through hydrogen-bonded phosphoric acid networks stabilized within the polymer matrix, enabling significant conductivity under anhydrous conditions [16,17].

This transition from hydration-controlled to acid-network-mediated transport fundamentally redefines membrane design variables. While PFSA systems are governed by water uptake and microphase morphology, PA–PBI membranes are controlled by ADL, proton transfer activation energy, and structural confinement of the polymer backbone. Thus, the shift to high-temperature operation represents not only a change in operating conditions, but a transformation in the underlying transport mechanism and design philosophy.

To better frame the transition from conventional hydrated PEM systems to high-temperature phosphoric acid-doped PBI membranes, Table 1 summarizes the main differences between PFSA and PA–PBI membranes in terms of transport medium, operating conditions, and dominant design constraints.

**Figure 2:**
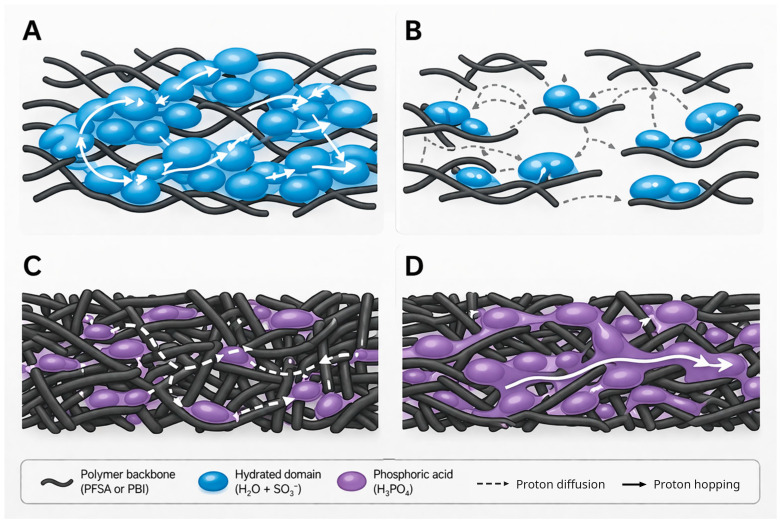
Transition from hydration-dependent proton transport in PFSA membranes to anhydrous acid-network conduction in phosphoric acid-doped PBI systems. (**A**) Fully hydrated PFSA morphology with percolated ionic domains supporting efficient water-mediated proton transport. (**B**) Disruption of percolation pathways at low relative humidity, resulting in a sharp decrease in conductivity, dashed arrow: proton diffusion. (**C**) Localized proton transport in PA–PBI membranes at low-to-intermediate ADL, where phosphoric acid is confined within the rigid polymer matrix and transport occurs via short-range hopping, dashed arrow: proton diffusion. (**D**) Formation of a percolated hydrogen-bonded H_3_PO_4_ network at high ADL, enabling long-range proton transport under anhydrous high-temperature conditions.

The mechanistic transition from hydration-dependent proton transport in PFSA membranes to acid-network-mediated conduction in PA–PBI systems is schematically illustrated in Figure 2.

This review aims to define the governing structure–property–performance relationships in PA–PBI membranes and to identify the key design variables required to simultaneously achieve high proton conductivity, mechanical stability, and long-term durability. By framing these parameters within a multidimensional design space, the work seeks to move beyond isolated material optimization toward a more predictive framework for membrane engineering.

### 1.2. Emergence of Phosphoric Acid-Doped Polybenzimidazole

Polybenzimidazole (PBI) emerged as one of the most robust candidates for high-temperature polymer electrolytes due to its exceptional thermal stability, rigid aromatic backbone, and chemical resistance. Its fully aromatic heterocyclic structure confers a glass transition temperature exceeding 400 °C [18], enabling structural integrity well beyond the operating range of HT-PEM fuel cells.

A defining feature of PBI is the presence of benzimidazole moieties along the polymer chain. These units contain basic nitrogen sites capable of forming strong acid–base complexes with phosphoric acid (H_3_PO_4_), which constitute the chemical foundation of PA–PBI membranes. Upon acid doping, PBI transitions from an electronically insulating material to a proton-conducting medium in which acid molecules are partly coordinated to the polymer backbone and partly organized into hydrogen-bonded clusters.

The key conceptual advance enabling HT-PEM operation was the recognition that PA–PBI membranes can sustain significant proton conductivity in the absence of bulk water. Proton transport proceeds through extended hydrogen-bonded phosphoric acid networks stabilized within the polymer matrix [8,16]. In contrast to PFSA membranes, where hydrated ionic domains define transport pathways, PA–PBI systems rely on acid–acid and acid–polymer interactions to establish percolated conduction pathways under anhydrous conditions.

Within this framework, the ADL becomes the central variable governing transport. Systematic studies have shown that conductivity increases strongly with ADL under anhydrous conditions [19,20,21]. At ADL ≈ 5–6 mol·RU^−1^, conductivity at 160 °C is ~0.02 S·cm^−1^, while increasing ADL to ≈10–11 mol·RU^−1^ raises σ to ~0.10–0.14 S·cm^−1^ [18]. These values, achieved without external humidification, marked a critical step toward practical HT-PEM operation.

Unlike PFSA membranes, where conductivity collapses upon dehydration, PA–PBI systems decouple proton transport from water activity. Instead, conductivity becomes governed by acid population and polymer structure. As ADL increases, the system transitions from isolated acid–base complexes to percolated hydrogen-bonded networks, enabling long-range proton transport. However, this transition also alters the mechanical state of the polymer.

Thus, PA–PBI membranes represent a paradigm shift in electrolyte design. The dominant control variables move from water uptake and microphase morphology (PFSA systems) to acid doping level, proton transfer energetics, and structural confinement. As a result, membrane optimization requires balancing proton conductivity with mechanical stability and acid retention.

Importantly, increasing ADL enhances proton mobility but also induces plasticization, swelling, and acid redistribution [18,22,23]. The success of PA–PBI membranes therefore depends on architectural strategies that enable access to high-conductivity regimes while preserving structural integrity. In this sense, phosphoric acid-doped PBI does not resolve the transport–stability trade-off, but instead defines a multidimensional design problem that underpins the remainder of this review.

### 1.3. Expanding the Design Space: Beyond Simple Acid Doping

**Figure 3:**
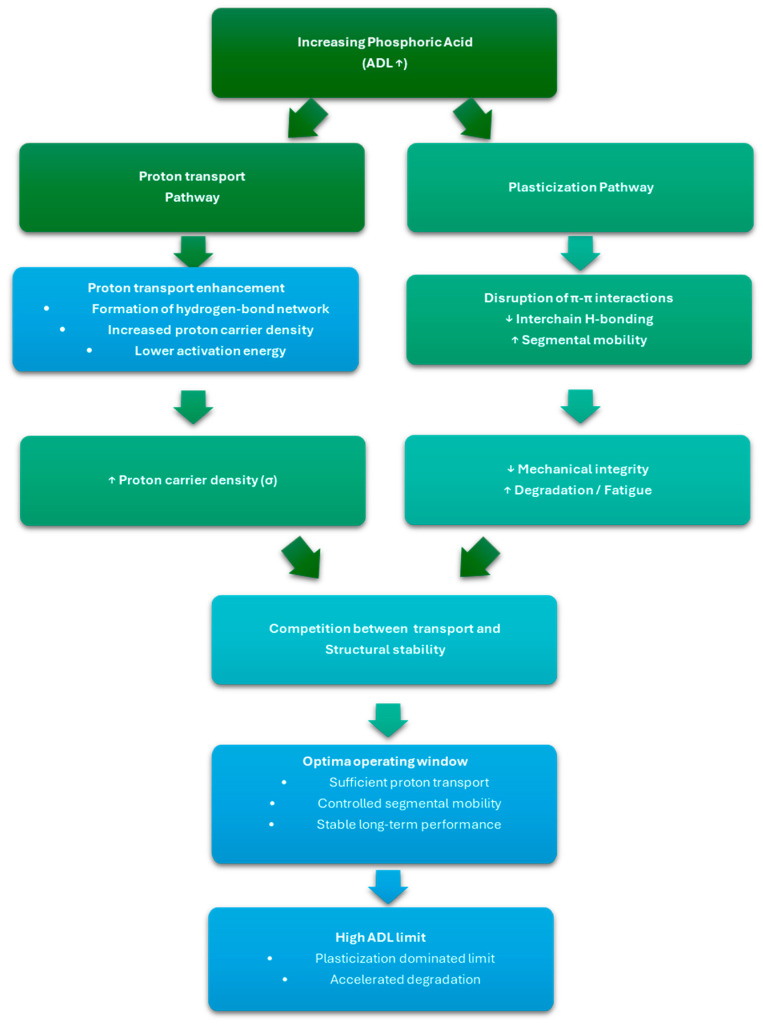
Flow diagram illustrating the dual role of phosphoric acid in PA–PBI membranes. Increasing acid content enhances proton transport through the formation of hydrogen-bond networks, while simultaneously promoting plasticization by increasing segmental mobility and weakening interchain interactions. The competition between these effects defines an optimal operating window and establishes the limits of segmental mobility and long-term durability.

Phosphoric acid therefore plays a dual role as both proton carrier and plasticizer, defining the central materials challenge in PA–PBI systems. **This dual role, and the resulting competition between proton-transport enhancement and plasticization-driven structural degradation, is schematically summarized in Figure 3**.

Representative target ranges can be identified from the literature. For stable high-temperature operation, proton conductivity typically lies in the range of ~0.1–0.2 S·cm^−1^ at 140–180 °C, while maintaining an acid doping level (ADL) sufficient to sustain a percolated hydrogen-bond network without inducing excessive plasticization. At the same time, mechanical integrity generally requires tensile modulus values on the order of several hundred MPa to prevent creep and structural degradation under operating conditions.

These ranges can be further interpreted in terms of practical limits. Below a critical ADL, the hydrogen-bond network remains discontinuous, leading to transport-limited behavior. Conversely, beyond a threshold ADL, excess phosphoric acid induces plasticization, increasing segmental mobility and accelerating mechanical degradation. Similarly, the mechanical modulus must remain sufficiently high to avoid creep and structural instability under operating conditions.

Together, these constraints define the hard boundaries of the design space, separating transport-limited, optimal, and degradation-dominated regimes.

In contrast to hydrated PFSA membranes, where conductivity and mechanical stability are largely governed by water content and microphase morphology, transport in PA–PBI systems is intrinsically coupled to polymer–acid interactions. The formation of extended phosphoric acid networks enhances proton mobility while reducing cohesive energy density, making conductivity and mechanical softening inseparable.

This coupling is clearly illustrated by molecular-weight-controlled studies. A high-molecular-weight PBI membrane (78 kDa) at ADL = 10.8 mol·RU^−1^ achieves σ ≈ 0.14 S·cm^−1^ at 160 °C while maintaining relatively stable operation (voltage decay ≈ 1.5 μV·h^−1^). In contrast, a lower-molecular-weight analogue (54 kDa) at comparable ADL exhibits a decay rate exceeding 100 μV·h^−1^ under identical conditions [20]. Despite similar conductivity, the degradation behavior differs by nearly two orders of magnitude, demonstrating that structural stability, not σ alone, controls device performance.

Recognizing this constraint has expanded the design space of PA–PBI membranes beyond simple ADL optimization. For example, Spasov et al. [24] demonstrated molecular-level control of pore architecture in PBI membranes through the incorporation of porous aromatic frameworks (PAFs), enabling simultaneous enhancement of mechanical properties and phosphoric acid retention. The introduction of micro- and mesoporous domains within the polymer matrix creates additional transport pathways while restricting polymer chain mobility, resulting in increased stiffness and reduced acid leaching, thereby illustrating how topology engineering can balance conductivity and structural stability. Multiple architectural strategies have been developed to mitigate plasticization while preserving proton transport. Crosslinking restricts chain mobility and suppresses swelling, enabling σ values up to ~0.25 S·cm^−1^ under optimized conditions [25,26,27,28]. However, excessive crosslinking can reduce segmental flexibility below the threshold required for effective proton hopping, limiting the degrees of freedom of the hydrogen-bond network. This leads to a non-monotonic relationship between crosslinking density and conductivity, with an optimal window beyond which transport is hindered and mechanical brittleness may emerge. Multiple architectural strategies have been developed to mitigate plasticization while preserving proton transport. Crosslinking restricts chain mobility and suppresses swelling, enabling σ values up to ~0.25 S·cm^−1^ under optimized conditions [25,26,27,28]. Backbone modification (e.g., para-ordering or fluorination) alters cohesive energy density and packing efficiency [18,29], while branching and star-like architectures introduce geometric confinement without permanent junctions.

A complementary approach focuses on energetic stabilization of proton hopping. Nitrogen-positive engineered membranes achieve σ ≈ 0.185 S·cm^−1^ at 180 °C with low activation energies (E_a_ ≈ 13–16 kJ·mol^−1^) [30], demonstrating that transport can be enhanced by lowering the proton transfer barrier rather than increasing acid population.

Hybrid architectures further illustrate the multidimensional nature of this design space. Imidazole-functionalized heteropolyacid composites reach σ ≈ 0.1666 S·cm^−1^ at 200 °C but suffer significant acid loss (~49–54%) under steam exposure [31]. Similarly, coordination-induced free-volume expansion increases ADL and σ but reduces tensile strength from ~75 MPa to ~35–45 MPa [29]. These examples highlight that conductivity gains achieved through increased acid content or morphological expansion often come at the expense of mechanical stability.

Collectively, these results define a multidimensional design space governed by:

• acid doping level (carrier density),
• activation energy for proton hopping,
• backbone rigidity and packing,
• crosslinking or topological confinement,
• mechanical resilience under operating conditions,
• acid retention over time.

From this perspective, the design of next-generation HT-PEM membranes can be described in terms of a limited set of governing descriptors. These include the acid doping level (ADL), which defines proton carrier density; the activation energy (E_a_), which reflects proton transfer efficiency within the hydrogen-bond network; the mechanical modulus, which determines resistance to plasticization; and structural descriptors such as free volume and topological confinement, which control transport pathways and stability. Together, these variables define the multidimensional scaling relations linking conductivity, mechanical integrity, and durability, providing a unified framework for a more predictive membrane design.

In contrast to PFSA systems, where performance is primarily controlled by hydration and ionic morphology, HT-PEM membranes must be engineered within a fully coupled transport–mechanical–durability framework.

Accordingly, this review treats PA–PBI membranes not as a simple doping continuum, but as a structured design space in which conductivity, mechanical stability, and retention are intrinsically linked. Section 2 analyzes the transport physics governing ADL and activation energy, Section 3 examines architectural strategies, Section 4 and Section 5 address chemo-mechanical coupling and acid retention, and Section 6 evaluates device-level performance.

This framework enables the identification of quantitative design rules that reconcile high proton conductivity with long-term stability, moving toward predictive membrane engineering.

Conceptually, this trade-off can be represented as a conductivity–stability relationship as a function of ADL. At low ADL, conductivity is limited but structural integrity is high. At intermediate ADL, an optimal operating corridor emerges where percolated hydrogen-bond networks enable high σ while maintaining sufficient mechanical stability. At high ADL, further increases in acid content lead to plasticization, mechanical degradation, and accelerated performance decay. While advanced mechanical characterization at elevated temperatures (e.g., DMA or creep testing) is essential to fully capture thermo-mechanical behavior under operating conditions, the present review focuses on trends reported in the literature, which are predominantly derived from room-temperature measurements.

To unify the diverse synthetic approaches, Table 2 maps the main strategies onto key design descriptors, including acid doping level (ADL), activation energy (E_a_), and mechanical properties, highlighting their associated trade-offs within the multidimensional design space.

### 1.4. Gaps in the Existing Literature

Despite numerous reports of incremental improvements in proton conductivity (*σ*) and peak power density ($P_{max}$), the PA–PBI literature has often treated transport, mechanical stability, and acid retention as only partially connected optimization targets. Several studies have linked subsets of the relevant variables in PA–PBI systems, including acid doping, proton conductivity, swelling, mechanical stability, acid distribution, and retention. However, these relationships have more often been examined in a partial or application-specific manner than within an explicit unified structure–property–durability framework. Many studies focus on enhancing σ through higher acid doping levels, electronic modification, or hybrid architectures, whereas others prioritize dimensional and mechanical stability through crosslinking, molecular-weight control, or topological design. As a result, the field contains many valuable advances, but fewer integrative analyses capable of comparing transport gains against their mechanical and durability costs.

Existing reviews of PBI-based membranes [16,31] typically classify materials according to synthetic route, crosslinking chemistry, or composite design. While such classifications are useful, they can obscure the deeper physical couplings among transport energetics, structural confinement, plasticization, acid mobility, and long-term electrochemical behavior. As discussed above, conductivity enhancement is rarely an isolated effect: increasing proton transport often coincides with changes in cohesive energy density, free volume, acid distribution, and membrane softness. Nevertheless, many studies still report conductivity improvements without corresponding mechanical, retention, or durability data, making cross-system comparison difficult and limiting the extraction of general design principles.

A similar fragmentation is evident in acid-retention and lifetime studies. Acid retention is evaluated under highly diverse and often non-comparable conditions, including steam exposure, open-circuit aging, and galvanostatic operation, whereas voltage decay rates (dV/dt) are reported across different temperatures, current densities, and testing protocols. This lack of harmonization makes direct comparison between architectures such as electronically stabilized membranes [30], hybrid imi-HPA systems [32], and high-molecular-weight PBI systems [18] inherently difficult. In many cases, retention, conductivity, and durability are each discussed within the same study, but not yet interpreted within a single common analytical framework.

This fragmentation leaves several important questions unresolved. First, what defines the upper conductivity limit under strictly anhydrous HT-PEM conditions? Reported values approach 0.18–0.20 S cm^−1^ in optimized systems [30], with even higher values in some composite or crosslinked membranes [26,33,34,35]. However, it remains unclear whether these values reflect an intrinsic transport ceiling of hydrogen-bonded phosphoric-acid networks or instead represent a practical limit imposed by concurrent mechanical weakening and acid mobility.

Second, how does acid mobility scale with acid doping level (ADL) and network topology? Increasing ADL enhances proton-carrier density, but it also increases the fraction of mobile acid species prone to redistribution and loss. Free-volume expansion strategies may increase both ADL and σ, but can also accelerate softening and plasticization [29], whereas electronic stabilization can reduce activation energy and improve retention without requiring extreme doping [30]. A broader framework linking acid mobility to topology, confinement, and transport energetics remains underdeveloped.

Third, which architectural strategies most effectively balance conductivity, mechanical stability, and retention? High-molecular-weight PBI, crosslinked networks, branched topologies, and hybrid systems each occupy different regions of the conductivity–mechanical–retention design space [18,36,37,38], yet direct comparisons under consistent conditions remain scarce. This makes it difficult to distinguish architecture-specific advantages from differences arising simply from testing conditions or reporting conventions.

Finally, what quantitative design rules can guide next-generation PA–PBI membranes? The field has progressed well beyond proof-of-concept demonstrations, but predictive relationships linking ADL, activation energy ($E_{a}$), swelling, mechanical properties, acid retention, and voltage decay are still insufficiently developed. Without such integrated criteria, progress remains largely empirical, case-specific, and difficult to generalize across membrane classes.

This review addresses these gaps by integrating transport physics, molecular architecture, mechanical stability, and device-level durability into a unified analytical framework. Rather than classifying PA–PBI membranes solely by chemistry or preparation route, we interpret them within a multidimensional design space that explicitly couples σ, $E_{a}$, structural confinement, plasticization, acid retention, and lifetime-related metrics.

By adopting this perspective, we aim to move beyond incremental optimization toward predictive design principles capable of defining the stable operating envelope of high-temperature polymer electrolytes.

### 1.5. Scope and Organization of This Review

This review adopts a mechanism-driven and quantitatively benchmarked perspective on phosphoric acid-doped PBI membranes for high-temperature PEM fuel cells. Rather than cataloguing materials by synthetic route or incremental performance gains, we integrate structural design strategies with transport energetics, chemo-mechanical stability, and durability within a unified analytical framework.

**Figure 4:**
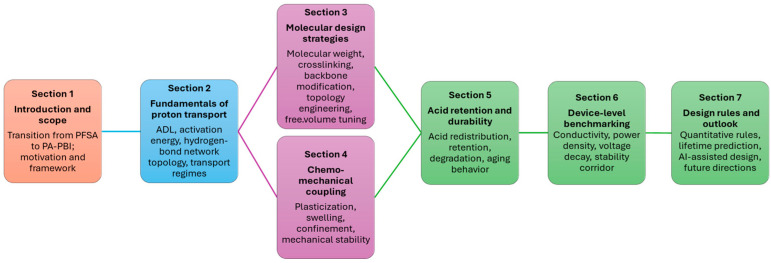
Flow chart summarizing the logical structure of the review. The diagram illustrates the progression from transport fundamentals (Section 2) to molecular design strategies (Section 3), chemo-mechanical coupling (Section 4), acid retention and durability (Section 5), device-level performance (Section 6), and the final design rules and outlook (Section 7).

The overall structure of the review and the relationships among the seven sections are summarized in Figure 4.

The central premise is that proton conductivity, mechanical stability, and acid retention cannot be optimized independently. Instead, they form a coupled system governed by ADL, activation energy (E_a_), network topology, and mechanical confinement. By linking these variables across molecular, mesoscale, and device levels, this review positions membrane architectures within a multidimensional performance landscape.

Section 2 establishes the physicochemical basis of proton transport in PA–PBI membranes, focusing on acid–base interactions, hydrogen-bond network formation, and the role of activation energy under anhydrous conditions. Section 3 examines molecular design strategies, molecular weight control, crosslinking, backbone tuning, topological engineering, and free-volume effects, and compares their quantitative impact on conductivity, mechanical properties, and transport energetics.

Section 4 addresses chemo-mechanical coupling, identifying plasticization as the primary origin of structural degradation in highly doped systems. Section 5 extends this analysis to acid retention and lifetime, integrating retention data, aging protocols, and voltage decay metrics. Section 6 benchmarks device-level performance within a conductivity–power–stability framework, highlighting the interplay between membrane properties and electrochemical operation.

Finally, Section 7 synthesizes these insights into quantitative design rules and outlines future directions for achieving simultaneously high conductivity, mechanical robustness, and long-term durability.

By integrating transport physics, structural design, and electrochemical stability, this review moves beyond reporting record conductivity values toward a predictive framework for PA–PBI membrane engineering. The goal is not only to maximize σ or P_max, but to define the structural and energetic conditions required to sustain performance over technologically relevant timescales. For clarity, Table 3 summarizes the main abbreviations used throughout this review.

## 2. Fundamentals of Proton Transport in PA–PBI Membranes

### 2.1. Acid–Base Complexation and Definition of Transport Regimes

Proton transport in PA–PBI membranes originates from acid–base complexation between benzimidazole nitrogen sites and phosphoric acid (H_3_PO_4_). The imidazole groups act as proton acceptors, forming strong hydrogen bonds that anchor a fraction of acid molecules directly to the polymer backbone, while the remaining acid organizes into hydrogen-bonded clusters. The resulting structure consists of a dynamic network of acid species embedded within a rigid aromatic matrix.

The key parameter governing this structure is the ADL. ADL controls both proton carrier density and the relative fraction of bound versus mobile acid species, thereby determining the topology of the hydrogen-bond network.

In contrast to PFSA membranes, where proton transport relies on hydration-dependent ionic channels, PA–PBI systems operate through hydrogen-bonded phosphoric acid networks that remain active under anhydrous conditions. As a result, conductivity is decoupled from water activity and becomes primarily governed by ADL and polymer structure.

A strong dependence of conductivity on ADL is consistently observed in high-molecular-weight PBI membranes under anhydrous conditions [18]. At ADL ≈ 5–6 mol·RU^−1^, σ at 160 °C is ~0.02 S·cm^−1^, while increasing ADL to ≈10–11 mol·RU^−1^ raises conductivity to ~0.10–0.14 S·cm^−1^. Under optimized conditions, σ values approach ~0.14–0.18 S·cm^−1^ in this regime. Representative proton transport and acid-doping data for PA–PBI membranes and benchmark systems are summarized in Table 4.

This evolution reflects a transition from isolated acid–base complexes at low ADL to percolated hydrogen-bonded networks at higher doping levels. While increasing ADL enhances carrier density and network connectivity, it also increases the fraction of mobile acid species. Consequently, conductivity gains are intrinsically coupled to mechanical softening and durability limitations, as discussed in subsequent sections.

**Table 4:** Proton transport and doping characteristics (PA–PBI and benchmark systems).

Source	Type	MW	ADL	AU	σ	T	Ea	Notes
		kDa		wt% or %	S·cm^−1^	°C	kJ/mol	
[18]	High-MW PBI (PBI-78 kDa/10.8 PA)	78	10.8	–	0.14	160	–	High-MW baseline; high ADL sustained
[30]	AmPBI-PIL (CTDTr) crosslinked, N+ site engineered	–	–	PA uptake 574.3% (CTDTr); 455.5–467.6% (others)	0.1849	180	13–16	Retention: 83% @160 °C after 240 h; Pmax 550.9 mW/cm^2^ @160 °C (H_2_/O_2_)
[32]	Arylether-type PBI/imi-HPA composite	–	–	290.4% (uptake)	0.1666	200	–	Acid loss ~49–54% after steam exposure
[29]	Cavity-engineered o-PBI (Zn/Co coordination route)	–	7.06 (neat); 9.54 (Zn); 9.76 (Co)	PA uptake 173/234/239 (neat)/(Zn);/(Co)	0.126 (Zn); 0.111 (Co); 0.068 (neat)	160	39–47	Free volume 42.72% → 57.05% (Zn)/56.7% (Co); cavity size ~8 Å; tensile 75 → 45/35 MPa
[31]	Crosslinked PBI systems	–	var.	–	≤0.25 (range)	160–180	–	Benchmark upper range; details depend on subtype
[32]	Crosslinked QPAES + OA-POSS (benchmark non-PBI)	–	11.6	–	0.0974	180	–	Pmax 461 mW/cm^2^ @200 °C
[32]	Ph-PBI/imi-HPA-3–15% composite (imidazole-substituted heteropolyacid salts)	Mn 58; Mw 90 (Đ 1.55)	–	ADLs 290.4% (72 h, 15 wt% imi-HPA-3)	0.1666 (200 °C, anhydrous)	200 (σ); 160 (cell)	–	MEA: Pt ~0.6 mg/cm^2^; gases: H_2_ 0.3 L/min, O_2_ 0.15 L/min; thickness 80 μm.

The evolution of proton transport with ADL can be described in terms of three progressively connected regimes (Figure 2).

At low ADL (≤2 mol·RU^−1^), proton transfer occurs primarily between imidazole nitrogen sites and tightly coordinated phosphoric acid molecules. The acid population is insufficient to form extended hydrogen-bond networks, and transport remains localized. Proton mobility is therefore limited by the availability of discrete hopping sites and relatively high activation barriers.

At intermediate ADL (≈3–6 mol·RU^−1^), additional acid molecules form short hydrogen-bond chains between H_2_PO_4_^−^ species, partially bridging adjacent polymer segments. This increases proton mobility while maintaining strong coupling between acid and backbone. Conductivity rises significantly compared to the low-ADL regime but remains sensitive to polymer confinement.

At high ADL (≥8–10 mol·RU^−1^), percolated hydrogen-bonded networks emerge, enabling long-range proton transport along interconnected H_2_PO_4_^−^ and H_3_PO_4_ species. This regime corresponds to the sharp increase in σ observed experimentally and marks the onset of technologically relevant conductivity.

Importantly, this percolation transition also increases the fraction of mobile acid species, linking enhanced transport to mechanical softening and retention limitations. Thus, ADL simultaneously defines the threshold for network connectivity and the onset of structural instability.

This coupling between carrier density and mechanical destabilization represents the fundamental constraint governing PA–PBI membrane performance. Subsequent sections examine how molecular architecture and transport energetics modulate this balance.

### 2.2. Energetic Landscape: Activation Energy and Proton Hopping

Beyond carrier density, the activation energy for proton transfer ($E_{a}$) provides direct insight into the dominant transport mechanism in PA–PBI membranes. While ADL governs the population of proton-conducting species, $E_{a}$ determines the efficiency of proton hopping along hydrogen-bonded networks. Arrhenius analyses show that $E_{a}$ varies widely with molecular architecture, acid–polymer interactions, and network topology.

In electronically engineered membranes featuring nitrogen-positive sites, σ≈0.185 S·cm^−1^ was achieved at 180 °C with low activation energies ($E_{a}$ ≈ 13–16 kJ·mol^−1^) [30]. These values are among the lowest reported for PA–PBI systems under anhydrous conditions and are consistent with highly efficient Grotthuss-type proton transport. Electrostatic interactions between positively charged nitrogen sites and H_2_PO_4_^−^ anions stabilize proton-transfer transition states, lowering the free-energy barrier for hopping.

Importantly, these low $E_{a}$ values are achieved without extreme increases in ADL, demonstrating that conductivity can be enhanced through energetic stabilization rather than increased carrier density. This distinction highlights two complementary pathways for improving σ: increasing proton population (via ADL) or reducing the hopping barrier (via $E_{a}$).

In contrast, coordination-induced cavity systems [29] illustrate the limitations of purely geometric approaches. Although ADL increases (from ∼7 to ∼9.5 mol·RU^−1^) and σ rises accordingly, activation energies remain high (≈39–47 kJ·mol^−1^). This indicates that increased carrier density alone does not guarantee efficient transport if proton-transfer energetics remain unfavorable. Fluorinated backbone variants further demonstrate the decoupling between ADL and energetic control. Reported activation energies span 27.5–58 kJ·mol^−1^ depending on substitution pattern, indicating that chemical modification alters acid–polymer interactions and hydrogen-bond dynamics independently of carrier density. High-molecular-weight PBI systems typically occupy an intermediate regime, with extended hydrogen-bond networks at ADL ≈10–11 mol·RU^−1^ and moderate activation energies consistent with partially constrained proton hopping [18].

Taken together, these observations indicate that proton conductivity arises from two interdependent but distinct contributions: carrier density and transport efficiency. Conceptually,

$$\sigma \propto (\mathrm{carrier}\;\mathrm{density},\;\mathrm{governed}\;\mathrm{by}\;\mathrm{ADL})\times f(E_{a}^{-1})$$

ADL controls the number of available proton carriers and the extent of network formation, whereas $E_{a}$ governs the efficiency of proton transfer along these networks. Increasing ADL without reducing $E_{a}$ may lead to diminishing returns and increased mechanical instability, while lowering $E_{a}$ enables high conductivity at moderate doping levels.

In this context, the literature describes proton conductivity in PA–PBI membranes through several complementary quantitative formulations, depending on whether the emphasis is placed on experimental measurement, thermally activated transport, or structural proton diffusion. First, conductivity is often reported in its experimental geometrical form, obtained from impedance spectroscopy as σ=L/(B×D×R), where L is the electrode distance, B the membrane width, D the thickness, and R the measured ohmic resistance. This formulation is explicitly used, for example, in recent PA–PBI membrane studies to calculate through-plane conductivity from AC impedance data [39]. Second, proton conductivity is commonly analysed through an Arrhenius-type relation, written either as $ln\sigma =lnA-E_{a}/(RT)$ or equivalently as $\sigma T=Aexp(-E_{a}/RT)$, which highlights the thermally activated nature of proton migration and enables comparison of apparent activation energies across different PA–PBI systems [39,40]. Third, more mechanistic treatments relate conductivity to structural proton diffusion through a Nernst–Einstein-type formulation, thereby linking σ to the concentration of exchangeable protons and to the proton diffusion coefficient within the phosphoric-acid-rich hydrogen-bond network [41]. Taken together, these expressions show that proton conductivity in PA–PBI membranes is not described by a single universal law; rather, the literature expresses σ through complementary experimental, activated, and diffusion-based formulations, each emphasizing a different aspect of the coupled roles of temperature, acid content, and hydrogen-bond network organization.

This perspective defines a key design principle: activation energy is not a secondary parameter, but a primary lever for optimizing proton transport. Architectures combining moderate ADL with low $E_{a}$ achieve high conductivity with reduced plasticization risk, whereas high-ADL systems with elevated $E_{a}$ rely on excessive acid content to compensate for inefficient hopping.

The following sections examine how molecular architecture simultaneously influences activation energy, network topology, and mechanical stability in PA–PBI membranes.

### 2.3. Hydrogen-Bond Network Topology

The extended hydrogen-bonded phosphoric acid network formed at high ADL constitutes the structural backbone of proton conduction in PA–PBI membranes. While ADL determines carrier density (Section 2.1) and activation energy governs hopping efficiency (Section 2.2), the spatial organization of acid species ultimately controls whether continuous transport pathways can be sustained.

In high-molecular-weight PBI systems, increasing ADL leads to a transition from isolated acid–base complexes to percolated hydrogen-bond networks. As ADL approaches ~10–11 mol·RU^−1^, conductivity reaches ~0.10–0.14 S·cm^−1^ at 160 °C, reflecting the formation of continuous transport pathways. Beyond this percolation threshold, however, further increases in acid content primarily densify the network rather than improving connectivity, while simultaneously promoting plasticization and acid mobility. This behavior indicates the existence of a practical upper bound where transport is maximized but structural confinement begins to deteriorate.

Network topology can be further modified by introducing additional proton reservoirs. Hybrid imi-HPA composite membranes provide a representative example [32]. These systems achieve high acid uptake and σ ≈ 0.1666 S·cm^−1^ at 200 °C by incorporating heteropolyacid domains that act as proton-bridging nodes. From a topological perspective, the network evolves from polymer-anchored chains to a more interconnected hybrid structure.

However, this increased connectivity is accompanied by significant swelling (up to ~180%) and substantial acid loss (~49–54% under steam exposure), highlighting the trade-off between network extension and structural stability. Similarly, coordination-induced cavity systems [29] increase ADL and σ by expanding interchain spacing, but reduce tensile strength from ~75 MPa to ~35–45 MPa, reflecting weakened cohesive constraints.

These examples demonstrate that hydrogen-bond network topology must balance two competing requirements:

• sufficient percolation continuity to enable long-range proton transport,
• sufficient structural confinement to limit swelling and acid mobility.

From a percolation perspective, the conductivity increase observed near ADL ≈ 8–10 mol·RU^−1^ corresponds to the transition from disconnected clusters to a continuous network spanning the membrane. Beyond this point, additional acid primarily increases network density rather than connectivity, while progressively weakening the polymer matrix.

This balance defines a key design principle: optimal performance is achieved near the percolation threshold, where transport pathways are continuous but plasticization remains controllable. Subsequent sections examine how molecular architecture shifts this threshold and stabilizes the percolated network under operating conditions.

### 2.4. Morphology, Packing, and Transport Continuity

While ADL and activation energy define the chemical and energetic dimensions of proton transport, the spatial continuity of hydrogen-bond networks is strongly governed by polymer morphology. Backbone packing, chain order, branching, and free-volume distribution determine how acid clusters are distributed and whether percolation pathways remain mechanically supported.

Para-ordered PBI systems illustrate the role of backbone packing. Improved chain alignment and enhanced π–π interactions increase cohesive energy density relative to meta-oriented analogues, enabling σ ≈ 0.14–0.15 S·cm^−1^ at 180 °C at moderate ADL (~8 mol·RU^−1^) [18]. This indicates that tighter packing stabilizes hydrogen-bond networks at lower acid content, delaying the onset of plasticization by enhancing local confinement of acid species.

Topological engineering introduces an additional level of morphological control. Hyperbranched PBI architectures [42] achieve σ ≈ 0.168 S·cm^−1^ while maintaining tensile strengths of ~75–80 MPa, demonstrating that geometric confinement can simultaneously support proton transport and mechanical stability. Branching increases the number of junction points and redistributes acid clusters more uniformly, reducing the formation of large, weakly confined domains.

Star-like co-PBI systems [43] further extend this concept, with tensile strengths exceeding 100 MPa. These architectures suggest that structural redundancy and distributed stress pathways can expand the safe ADL window while preserving network continuity.

In contrast, free-volume expansion represents an opposing morphological strategy. Coordination-induced cavity systems [29] increase ADL and σ by enlarging interchain spacing, but reduce tensile strength from ~75 MPa to ~35–45 MPa. Here, enhanced percolation arises from increased acid accommodation, but at the cost of reduced mechanical confinement. The hydrogen-bond network becomes more extensive, yet less structurally supported.

These contrasting behaviors highlight that morphology regulates both proton mobility and mechanical stability. Tight packing and branching enhance confinement, enabling stable percolated networks at moderate ADL, whereas free-volume expansion increases connectivity but promotes plasticization and acid mobility.

Overall, proton transport in PA–PBI membranes is governed not only by carrier density (ADL) and hopping energetics (E_a_), but also by morphological architecture. The percolation threshold of the hydrogen-bond network is intrinsically linked to backbone packing and topology, which determine whether transport pathways can be sustained without mechanical degradation. This morphological dimension provides the structural basis for the molecular design strategies discussed in Section 3 and the chemo-mechanical coupling analyzed in Section 4.

### 2.5. Toward a Unified Transport Framework

The analysis presented in Section 2.1, Section 2.2, Section 2.3 and Section 2.4 supports a unified framework in which proton conductivity in PA–PBI membranes is governed by three coupled variables: ADL, activation energy for proton hopping (E_a_), and hydrogen-bond network topology.

Rather than acting independently, these parameters define a multidimensional transport space. Conceptually, conductivity can be expressed as:

σ ∝ f(ADL, E_a_, network topology)

where ADL determines proton carrier density, E_a_ governs hopping efficiency along hydrogen-bond networks, and topology defines both percolation continuity and mechanical confinement.

ADL controls the population of proton-conducting species and the extent of network formation. However, carrier density alone does not determine transport efficiency. Lowering E_a_ can significantly enhance σ even at moderate ADL, as demonstrated by electronically engineered systems [30], whereas increasing ADL without reducing E_a_ may yield diminishing returns.

Network topology provides the third dimension of control by regulating the spatial distribution and confinement of hydrogen-bonded pathways. Packing, branching, and crosslinking can stabilize percolation at moderate ADL, while free-volume expansion increases connectivity but often compromises mechanical integrity.

Importantly, these variables are interdependent. Increasing ADL can reduce effective E_a_ through improved connectivity, but also promotes plasticization and acid mobility. Reducing E_a_ can decrease the need for extreme doping, mitigating mechanical penalties. Modifying topology can shift the percolation threshold, but excessive confinement may hinder proton hopping.

As a result, high conductivity (σ ≈ 0.18–0.20 S·cm^−1^) can be achieved through distinct pathways, carrier-density enhancement, energetic stabilization, or topological optimization, each associated with specific trade-offs in mechanical stability and durability.

This framework provides a conceptual bridge between transport physics and molecular design. While Section 2 defines the governing variables, subsequent sections examine how architectural strategies manipulate ADL, E_a_, and topology to position membranes within a stable, high-performance region of the design space.

## 3. Molecular Design Strategies for Transport and Stability Enhancement

Enhancing proton transport in PA–PBI membranes cannot be decoupled from polymer architecture. As established in Section 2, conductivity (σ) arises from the interplay between ADL, E_a_, and hydrogen-bond network topology. However, the ability to access and sustain high-performance regimes without mechanical degradation is ultimately governed by structural design.

Increasing ADL enhances network connectivity and carrier density but also promotes plasticization and acid mobility. Similarly, lowering E_a_ through electronic stabilization improves transport efficiency, yet requires sufficient structural confinement to ensure durability. Polymer architecture therefore acts as a regulatory framework that constrains the transport variables defined in Section 2.

Over the past two decades, five principal design strategies have emerged to navigate this multidimensional optimization problem:

1. Molecular weight control, increasing entanglement density and suppressing creep without altering chemistry [18]
2. Crosslinking and network confinement, introducing junction points to restrict swelling while preserving percolation [31]
3. Backbone chemical modification, tuning cohesive energy density and acid–polymer interactions (e.g., para-ordering, fluorination) [18]
4. Topological engineering, using branching or star-like architectures to enhance confinement without permanent crosslinks [29]
5. Free-volume and coordination tuning, expanding acid accommodation to increase ADL, often at the expense of mechanical stability [29]

These strategies operate across multiple structural scales, from molecular-level substitution to mesoscale network topology, and influence transport and durability through distinct mechanisms. Importantly, none independently resolves the conductivity–stability trade-off; rather, each shifts the membrane within the multidimensional design space defined in Section 2.

From a transport perspective, molecular design modifies:

• the maximum sustainable ADL before plasticization dominates,
• the activation energy for proton hopping (E_a_),
• the continuity and confinement of the hydrogen-bond network,
• resistance to swelling and creep at elevated temperature.

From a durability perspective, architecture governs the membrane response to thermal stress, mechanical deformation, and acid redistribution during operation. As discussed in later sections, device-level performance and lifetime are determined by how effectively these factors are balanced.

The following subsections analyze these strategies comparatively, positioning each within the conductivity–mechanical–retention landscape established in Section 2.

### 3.1. Molecular Weight and Chain Entanglement

Molecular weight (MW) is one of the most effective and conceptually straightforward parameters for stabilizing PA–PBI membranes. Unlike chemical modification or crosslinking, MW does not alter acid–base interactions or the intrinsic transport mechanism, but instead increases entanglement density and mechanical resistance, thereby extending the operational window for high ADL.

Systematic studies demonstrate that only high-MW PBI membranes can sustain ADL values approaching 10–11 mol·RU^−1^ without severe degradation [18]. At comparable ADL, membranes with similar conductivity can exhibit dramatically different durability depending on MW. For example, high-MW systems operate in a low-decay regime, whereas lower-MW analogues show accelerated degradation under identical conditions.

This behavior highlights a key principle: MW does not directly enhance σ or reduce activation energy (E_a_), but stabilizes the hydrogen-bond network by constraining large-scale segmental motion. Increasing MW raises entanglement density, improving resistance to chain pull-out, creep, and macroscopic deformation. At high ADL, where acid-induced plasticization weakens interchain cohesion, entanglement becomes the dominant mechanism maintaining structural integrity.

From a design perspective, MW acts as a macrostructural confinement parameter. It enables access to high-ADL regimes by delaying the onset of plasticization without introducing additional chemical complexity. In the conductivity–mechanical landscape, increasing MW shifts membranes toward higher sustainable ADL while preserving tensile strength.

However, MW alone cannot fully resolve the transport–stability trade-off. It does not reduce E_a_ or strengthen acid–polymer interactions, and excessively high MW may introduce processing challenges such as increased viscosity and film inhomogeneity. As a result, MW engineering is most effective when combined with complementary strategies that address transport energetics or acid confinement.

Overall, molecular weight defines a baseline stabilization mechanism against which more advanced architectural modifications must be evaluated.

### 3.2. Crosslinking and Network Confinement

Crosslinking introduces permanent or semi-permanent junctions between polymer chains, limiting volumetric expansion and suppressing acid-induced swelling. In PA–PBI systems, where high ADL weakens interchain cohesion, crosslinking acts as an artificial reinforcement mechanism that constrains segmental mobility and mitigates plasticization. Unlike molecular weight control, which relies on entanglement statistics, crosslinking imposes defined chemical or ionic constraints at specific junction sites.

**Figure 5:**
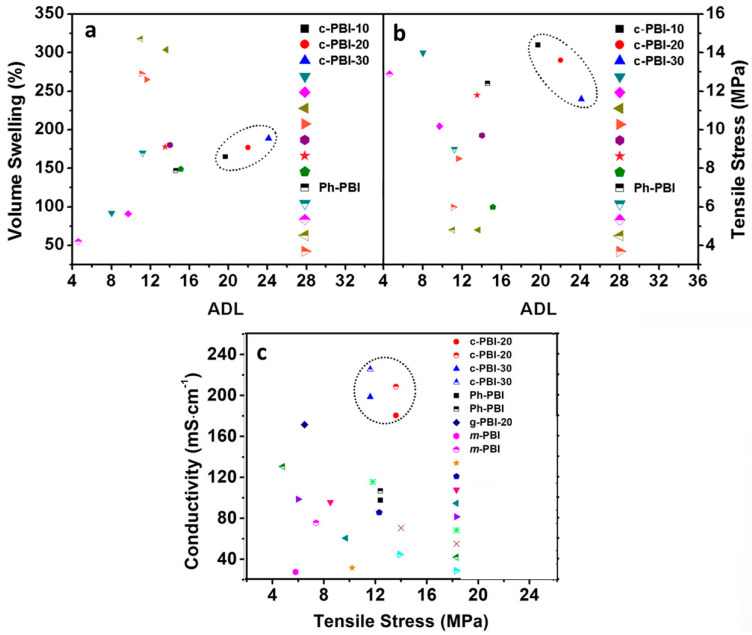
Effect of crosslinking on the transport–mechanical trade-off in PBI-based membranes: (**a**) volume swelling and (**b**) tensile strength as a function of ADL, and (**c**) relationship between proton conductivity and tensile strength. Crosslinked systems (filled symbols) exhibit improved mechanical stability at comparable conductivity levels relative to linear membranes. Adapted from ref. [26].

As illustrated in Figure 5, crosslinked PBI membranes can maintain high proton conductivity while exhibiting improved mechanical stability. This demonstrates that network confinement can extend the high-ADL operating window without fundamentally suppressing proton transport.

However, crosslinking introduces an intrinsic trade-off. Excessive crosslink density restricts local segmental motion and disrupts the dynamic rearrangement of hydrogen-bond networks required for efficient proton hopping. Within the framework of Section 2, this corresponds to an increase in the effective activation energy (E_a_) due to limited reorientation of H_2_PO_4_^−^ species. Thus, crosslinking must preserve network continuity while preventing large-scale deformation.

Early covalent crosslinking strategies improved dimensional stability and resistance to creep but often resulted in moderate conductivity (typically ~0.04–0.09 S·cm^−1^ at 140–160 °C) [44], reflecting this stiffness–transport trade-off. More advanced architectures, including hybrid or dual crosslinking approaches, demonstrate that properly tuned confinement can sustain higher ADL while maintaining competitive σ.

For example, POSS-assisted crosslinked membranes achieve high ADL and improved dimensional stability, although with reduced tensile strength relative to linear high-MW systems. More generally, the effectiveness of crosslinking depends strongly on crosslink density, distribution, and chemical nature. Ionic crosslinks may provide reversible confinement that preserves local mobility, whereas covalent crosslinks offer stronger stabilization but risk over-constraining the network.

Within the conductivity–mechanical design space, crosslinking shifts membranes toward higher sustainable ADL while maintaining moderate mechanical integrity. Unlike free-volume strategies, which enhance conductivity by increasing acid accommodation, crosslinking prioritizes structural confinement and dimensional stability.

The key design principle is therefore not maximal rigidity, but optimized crosslink density. Insufficient confinement permits swelling and acid redistribution, whereas excessive confinement disrupts hydrogen-bond percolation and increases the effective transport barrier. Optimal systems operate within a narrow window where macroscopic deformation is suppressed while local hydrogen-bond dynamics remain active.

Thus, crosslinking functions as a mesoscale confinement strategy that modifies the topological dimension of the transport framework. It does not alter the fundamental transport mechanism, but defines the mechanical boundary within which high-conductivity operation can be sustained.

### 3.3. Backbone Chemical Tuning and Packing Effects

Beyond molecular weight and crosslinking, chemical modification of the aromatic backbone provides a more subtle yet highly effective route to tune both proton transport and mechanical stability in PA–PBI membranes. Unlike MW and crosslinking, which act at the level of entanglement or network junctions, backbone chemistry directly modifies cohesive energy density, chain packing, and acid–polymer interaction strength at the molecular scale.

Para-ordered PBI systems exemplify this effect. Enhanced chain alignment and π–π stacking increase interchain cohesion relative to meta-oriented analogues, enabling σ ≈ 0.14–0.15 S·cm^−1^ at 180 °C at moderate ADL (~8 mol·RU^−1^) [18]. These systems achieve high conductivity with comparatively strong mechanical properties, indicating that improved packing stabilizes hydrogen-bond networks without requiring extreme acid loading.

Within the framework of Section 2, backbone ordering primarily modifies network topology by enhancing local confinement. In the conductivity–mechanical landscape, para-ordered systems shift toward regions where moderate-to-high σ coexists with improved tensile strength.

Fluorinated PBI derivatives further demonstrate the role of chemical substitution in tuning transport energetics. Reported activation energies span ~27–58 kJ·mol^−1^, while tensile strengths range from 55 to 111 MPa [45]. These variations indicate that backbone chemistry alters acid–polymer interactions and hydrogen-bond dynamics, enabling modulation of E_a_ independently of carrier density.

From an energetic perspective, chemical substitution modifies the strength and geometry of hydrogen bonds between phosphoric acid and benzimidazole sites. Changes in local polarity and steric environment influence proton-transfer transition states, providing a pathway to reduce E_a_ without increasing ADL.

Copolymers incorporating different benzimidazole motifs introduce an additional level of control by combining rigid and flexible segments within a single backbone [46]. This enables simultaneous tuning of packing density, acid affinity, and swelling behavior, improving durability while maintaining moderate conductivity.

In contrast to crosslinking or MW control, backbone chemistry acts as a continuous, fine-scale tuning parameter. It can:

• shift the percolation threshold of the hydrogen-bond network,
• modify activation energy (E_a_),
• enhance resistance to plasticization at moderate ADL,
• influence acid uptake and retention.

However, backbone rigidity alone cannot eliminate the transport–stability trade-off. Excessively rigid systems may hinder local hydrogen-bond reorganization and increase E_a_, while overly flexible systems can promote acid mobility and degradation.

Overall, backbone modification operates as a microstructural tuning mechanism within the multidimensional design space defined in Section 2 and Section 3. It does not alter the fundamental transport mechanism, but shifts both energetic and mechanical coordinates, enabling access to stable high-conductivity regimes. The following subsection examines how topological design and free-volume modulation further expand this design space.

### 3.4. Topological Engineering: Branched and Star Architectures

Topological modification introduces architectural confinement without relying on permanent covalent crosslinks. Whereas crosslinking imposes discrete junctions and molecular weight increases entanglement statistically, branching and star-like architectures modify chain distribution at the mesoscale. This geometric approach alters packing statistics, effective entanglement density, and acid distribution while preserving chemical continuity along the backbone.

Hyperbranched PBI membranes [42] exemplify this strategy, achieving σ ≈ 0.168 S·cm^−1^ while maintaining tensile strengths of ~75–80 MPa. Compared to highly doped linear systems, this combination of elevated conductivity and mechanical robustness highlights the effectiveness of geometric confinement.

Branching increases the density of chain ends and junction points, enhancing entanglement-like confinement without introducing rigid crosslinks. This redistributes mechanical stress and suppresses large-scale chain slippage under plasticization, while preserving sufficient segmental mobility for hydrogen-bond reorganization.

Within the framework of Section 2, topological engineering primarily modifies network topology. It does not directly alter ADL or E_a_, but shifts the structural boundary within which high ADL can be sustained. In the conductivity–mechanical landscape, branched systems move toward regions of high σ with retained mechanical stability.

Star-like co-PBI architectures [43] further extend this concept. With tensile strengths exceeding 100 MPa, these systems demonstrate exceptional resistance to deformation while maintaining proton-conducting pathways. Their dual topology, branched cores combined with linear arms, provides structural anchoring alongside extended hydrogen-bond connectivity, enabling a form of “soft confinement” distinct from rigid crosslinking.

Compared to free-volume expansion strategies (Section 3.5), which increase conductivity by enhancing acid accommodation, topological engineering improves mechanical resistance without significantly increasing the fraction of mobile acid species. As a result, branched systems tend to shift toward a balanced region of the conductivity–mechanical space, rather than approaching the plasticization boundary.

The distinction from crosslinking is therefore critical. Crosslinking introduces fixed junctions that may over-constrain local dynamics and increase effective E_a_ if excessive, whereas topological engineering reshapes chain packing without disrupting hydrogen-bond continuity. It modifies macroscopic deformation while preserving local transport pathways.

However, topological strategies are not without limitations. Excessive branching can increase synthetic complexity, polydispersity, and microstructural heterogeneity. Moreover, without complementary stabilization mechanisms, control of acid distribution and long-term retention remains challenging.

Overall, topological engineering represents a mesoscale confinement strategy that enhances mechanical stability while preserving proton transport. By modifying chain packing and effective entanglement, it expands the accessible design space for high-performance PA–PBI membranes. The following subsection examines the complementary approach of free-volume and coordination tuning, which enhances carrier density but introduces distinct stability challenges.

### 3.5. Free-Volume and Coordination Engineering

Coordination-induced cavity formation represents a complementary architectural strategy in which transient metal coordination expands interchain spacing prior to acid doping. Unlike crosslinking or backbone modification, which enhance structural confinement, this approach deliberately loosens chain packing to increase acid accommodation and promote hydrogen-bond network formation.

In coordination-modified PBI membranes [29], increased free volume raises ADL (from ~7 to ~9.5 mol·RU^−1^) and enhances conductivity (from ~0.068 to ~0.126 S·cm^−1^ at 160 °C), reflecting improved network percolation. However, this gain in σ is accompanied by significant mechanical degradation: tensile strength decreases from ~75 MPa to ~35–45 MPa, and activation energy remains relatively high (≈39–47 kJ·mol^−1^).

Within the transport framework of Section 2, free-volume expansion primarily increases carrier density (ADL) while leaving proton-transfer energetics largely unchanged. As a result, conductivity improvements arise from increased acid population rather than enhanced hopping efficiency.

This distinction becomes evident when compared to electronically engineered systems, where high σ is achieved through reduced E_a_ rather than increased ADL. In contrast, free-volume strategies require higher acid loading to compensate for relatively inefficient proton transfer.

In the conductivity–mechanical landscape, free-volume systems shift upward in σ but downward in tensile strength, approaching the plasticization boundary. The increased fraction of loosely confined acid species also enhances susceptibility to creep and acid redistribution, linking this strategy directly to retention limitations.

Mechanistically, reduced packing density increases segmental mobility and facilitates acid incorporation, but weakens resistance to deformation under operating conditions. Without sufficient confinement, the resulting hydrogen-bond network becomes more extensive yet less mechanically supported.

This leads to a key design insight: increasing carrier density alone does not optimize proton transport. Without concurrent reduction in E_a_ or sufficient structural confinement, gains in σ may be offset by reduced durability.

Nevertheless, free-volume engineering remains valuable when used in combination with stabilizing strategies. Controlled expansion, coupled with backbone rigidity or moderate crosslinking, can increase ADL without catastrophic mechanical degradation. In such cases, free volume acts as a fine-tuning parameter rather than a primary design lever.

Overall, coordination-induced free-volume expansion enhances conductivity through increased acid accommodation, but introduces significant mechanical and retention challenges. Its effectiveness therefore depends on integration with complementary energetic and structural stabilization strategies.

### 3.6. Comparative Architectural Landscape

Positioning the architectural strategies discussed in Section 3.1, Section 3.2, Section 3.3, Section 3.4 and Section 3.5 within the unified framework of Section 2 reveals clear and systematic trends. Rather than independent routes to conductivity enhancement, these strategies occupy distinct regions of a multidimensional design space defined by ADL, activation energy (E_a_), network topology, and mechanical confinement.

Molecular weight control and topological engineering primarily enhance mechanical resilience. By increasing entanglement density or geometric confinement, these approaches shift the plasticization boundary toward higher ADL, enabling stable operation at elevated acid content. However, their effect is predominantly structural, with limited influence on E_a_.

Crosslinking introduces fixed junctions that suppress swelling and macroscopic deformation, allowing membranes to operate at higher ADL. At the same time, excessive crosslink density may disrupt hydrogen-bond continuity and increase effective E_a_, resulting in a narrow optimal window between mobility and rigidity.

Backbone chemical modification acts at a finer scale, tuning cohesive energy density and acid–polymer interactions. This enables simultaneous adjustment of network topology and, in some cases, E_a_, improving transport efficiency without requiring extreme acid loading.

In contrast, free-volume and coordination strategies enhance conductivity primarily through increased carrier density. While these approaches increase ADL and network connectivity, they reduce mechanical confinement and typically maintain relatively high E_a_, leading to increased susceptibility to plasticization and degradation.

Electronic site engineering represents the most direct route to energetic optimization. By lowering E_a_, it enables high conductivity without excessive ADL, thereby reducing the mechanical penalties associated with high acid loading.

Taken together, these strategies demonstrate that no single architectural approach simultaneously maximizes conductivity, mechanical stability, and retention. High σ can be achieved through increased carrier density or reduced activation energy, but without sufficient structural confinement, durability is compromised. Conversely, strategies that maximize mechanical stability may limit transport efficiency if energetic barriers remain significant.

As a result, optimal performance is achieved within a constrained region of the design space, a “stability corridor”, characterized by:

• ADL ≈ 10–12 mol·RU^−1^,
• σ ≳ 0.14–0.18 S·cm^−1^ under anhydrous conditions,
• sufficient mechanical strength to suppress creep,
• controlled acid mobility to ensure retention stability.

Achieving this balance typically requires combining complementary design elements: moderate-to-high ADL for carrier density, structural confinement (via MW, branching, or controlled crosslinking), and energetic stabilization to reduce E_a_. Architectures relying on a single extreme parameter, either excessive ADL or excessive rigidity, tend to fall outside this optimal region.

Thus, PA–PBI membrane design should be interpreted not as maximizing individual performance metrics, but as positioning materials within a multidimensional transport–stability landscape. The following sections examine how this positioning governs chemo-mechanical coupling (Section 4), acid retention (Section 5), and device-level performance (Section 6).

As shown in Table 5, increasing acid doping level or introducing free-volume-enhancing strategies systematically reduces tensile strength and promotes swelling, confirming that conductivity enhancement is intrinsically coupled to mechanical softening.

## 4. Chemo-Mechanical Coupling and Plasticization as the Structural Origin of Durability Limits

The enhancement of proton conductivity in PA–PBI membranes is intrinsically coupled to mechanical softening induced by phosphoric acid. As discussed in Section 3, increasing ADL expands the accessible conductivity window, but simultaneously alters the mechanical state of the polymer matrix. This coupling between transport enhancement and structural weakening represents the fundamental limitation in high-temperature operation.

Phosphoric acid acts not only as a proton carrier but also as a plasticizer. Upon doping, acid molecules intercalate between aromatic chains, weakening π–π interactions and disrupting interchain hydrogen bonding. At low ADL, most acid remains strongly coordinated to benzimidazole sites, and the polymer backbone retains its structural integrity. At higher ADL (≈8–10 mol·RU^−1^ and above), an increasing fraction of loosely bound acid forms extended hydrogen-bonded networks, enabling efficient proton transport while simultaneously reducing cohesive energy density.

This transition leads to a progressive softening of the polymer matrix, characterized by increased segmental mobility, reduced tensile strength, and enhanced creep. As summarized in Table 5. Mechanical and dimensional stability metrics (plasticization envelope)., increasing acid content systematically reduces mechanical strength and promotes swelling. These effects are not secondary, but directly govern long-term electrochemical stability.

The dependence of mechanical stability on acid doping level can be described in a simplified semi-empirical form. Experimental studies consistently show that increasing ADL leads to a progressive reduction in tensile strength due to acid-induced plasticization [18,29]. This behavior can be approximated as:

$$S=S_{0}exp(-\alpha \cdot ADL)$$

where $S_{0}$ is the mechanical strength of the undoped polymer and α is an effective plasticization coefficient reflecting the strength of acid–polymer interactions. Although this expression is not a universal law, it provides a convenient phenomenological description of the exponential-like decay of mechanical properties with increasing acid content observed across different PA–PBI systems.

This trend is consistent with the morphological evolution of the hydrogen-bond network, where increasing ADL promotes network percolation while simultaneously reducing structural confinement.

Increasing membrane thickness may improve apparent mechanical robustness at the device scale by delaying failure or reducing the impact of local defects; however, it does not compensate for the intrinsic reduction in tensile strength and modulus induced by acid-driven plasticization.

Importantly, plasticization introduces a feedback mechanism linking structure and transport:

As ADL increases, cohesive interactions within the polymer matrix are progressively weakened, resulting in higher segmental mobility and, ultimately, increased acid mobility.

This feedback accelerates acid redistribution under operating conditions, contributing to conductivity loss and voltage decay. Consistent with this mechanism, membranes with reduced tensile strength exhibit higher degradation rates.

Experimental observations further indicate that plasticization imposes a practical upper bound on sustainable ADL. Near ADL ≈ 10–11 mol·RU^−1^, membranes achieve technologically relevant conductivity while retaining moderate mechanical stability. Beyond this regime, additional acid does not significantly increase σ, but instead accelerates mechanical degradation and performance loss.

Thus, chemo-mechanical coupling emerges as the governing constraint in PA–PBI membrane design. Strategies that enhance conductivity must therefore be evaluated in terms of their impact on structural resilience and resistance to acid redistribution. The following subsections examine how different architectural approaches modulate this coupling and delay the onset of plasticization-driven failure.

### 4.1. Plasticization Across Architectures

Quantitative evidence of chemo-mechanical coupling in PA–PBI membranes is most clearly demonstrated in molecular-weight-controlled systems [18]. Because molecular weight modifies chain entanglement density without altering chemical composition, these systems provide a controlled framework for isolating structural effects from purely chemical or energetic contributions.

At comparable ADL, membranes with similar conductivity can exhibit dramatically different durability depending on molecular weight. High-MW membranes operate in a low-decay regime, whereas lower-MW analogues show degradation rates differing by orders of magnitude under identical electrochemical conditions. This disparity highlights that durability is far more sensitive to mechanical integrity than to small variations in σ once hydrogen-bond network percolation is achieved.

Analysis of the data summarized in Table 2 and Table 3 indicates that the transition from stable to unstable operation occurs within a narrow tensile strength window. Above ~25–30 MPa (room temperature reference), membranes exhibit low voltage decay and stable performance. Below this threshold, creep and structural relaxation accelerate, leading to acid redistribution and increased ohmic resistance.

Membranes with higher tensile strength cluster in the low-decay regime, while mechanically weakened systems exhibit significantly higher dV/dt. The trend is systematic: increased segmental mobility leads to accelerated electrochemical degradation.

Mechanistically, this behavior reflects the role of entanglement density in constraining deformation under high ADL. In high-MW systems, the hydrogen-bond network remains percolated and structurally supported, limiting large-scale redistribution of acid species. In contrast, lower-MW systems allow greater segmental motion under thermal and electrochemical stress, facilitating local acid depletion near catalyst interfaces and increasing interfacial resistance.

These results demonstrate that mechanical stability is a primary determinant of electrochemical lifetime in PA–PBI membranes. Once conductivity reaches the technologically relevant regime (σ ≈ 0.10–0.14 S·cm^−1^), further gains do not compensate for mechanical weakening. Instead, durability becomes governed by structural resilience.

Thus, molecular-weight-controlled systems provide direct quantitative validation of the chemo-mechanical coupling framework introduced in Section 2. They establish that the stability of hydrogen-bond networks depends not only on connectivity but also on the mechanical integrity of the surrounding polymer matrix, providing a benchmark for evaluating alternative stabilization strategies.

### 4.2. Backbone Order and Cohesive Energy Density

Backbone chemistry modifies plasticization resistance independently of molecular weight and crosslink density by tuning cohesive energy density at the molecular scale. Whereas molecular weight enhances entanglement statistically, chemical modification alters interchain interactions, directly influencing how the polymer framework responds to acid intercalation.

Para-ordered PBI systems provide a representative example. Improved chain alignment and enhanced π–π stacking increase interchain cohesion relative to meta-oriented analogues, enabling σ ≈ 0.14–0.15 S·cm^−1^ at 180 °C at moderate ADL (~8 mol·RU^−1^) [18]. At the same time, tensile strengths in the range of 33–58 MPa indicate that relatively high conductivity can be achieved without severe mechanical softening.

This behavior reflects enhanced packing efficiency, which stabilizes hydrogen-bond networks within a mechanically supported environment. Compared to linear systems, para-ordering enables comparable conductivity at lower ADL, effectively delaying the onset of plasticization.

Fluorinated PBI derivatives [45] further illustrate the role of chemical substitution. Reported tensile strengths of 55–111 MPa indicate significantly increased rigidity, while variations in activation energy suggest that fluorination modifies acid–polymer interactions and local hydrogen-bond dynamics. In this way, backbone chemistry can influence both mechanical stability and proton-transfer energetics without relying solely on increased acid loading.

From a chemo-mechanical perspective, these modifications enhance resistance to creep and structural relaxation under operating conditions. However, backbone rigidity alone does not eliminate plasticization. As ADL increases beyond the percolation threshold, all systems experience some degree of softening. Increased rigidity instead reduces the rate at which mechanical degradation occurs, thereby extending the stable operating window.

Thus, backbone chemical modification acts as a microstructural regulator of the plasticization boundary. It does not alter the fundamental transport mechanism, but shifts the balance between conductivity and mechanical stability by increasing cohesive energy density.

Compared to molecular-weight engineering (Section 4.1), which relies on entanglement density, backbone tuning provides intrinsic rigidity. Compared to crosslinking (Section 4.3), it avoids introducing rigid junctions that may hinder hydrogen-bond reorganization. As such, backbone chemistry represents a fine-scale stabilization strategy that improves durability without fundamentally altering transport pathways.

The following subsection examines how confinement strategies at larger structural scales further modify this balance

### 4.3. Crosslinking: Constraining Macroscopic Swelling

Crosslinking introduces inter-chain constraints that limit volumetric expansion under high acid loading. Unlike molecular weight, which enhances entanglement statistically, crosslinking imposes discrete junction points that restrict large-scale chain displacement. In PA–PBI membranes, this mesoscale confinement becomes particularly important once ADL exceeds the percolation threshold.

Comprehensive studies report that crosslinked PBI systems can achieve conductivities approaching 0.25 S·cm^−1^ at 160–180 °C under optimized conditions [31], demonstrating that network confinement does not inherently suppress proton transport. Rather, when appropriately tuned, crosslinking enables operation at high ADL while mitigating swelling and creep.

In practice, crosslink density and distribution strongly influence the transport–mechanical balance. For example, POSS-assisted crosslinked membranes [47] achieve ADL ≈ 11.6 mol·RU^−1^ and σ ≈ 0.0974 S·cm^−1^ at 180 °C, while delivering peak power densities of ~461 mW·cm^−2^ at 200 °C. Although tensile strength in these systems remains lower than in high-MW linear analogues, dimensional stability under high-temperature operation is significantly improved.

From a structural perspective, crosslinking reduces free-volume fluctuations and restricts cooperative segmental motion. This suppression of macroscopic deformation slows acid redistribution and mitigates creep-driven degradation, consistent with the lower voltage decay rates observed in mechanically stabilized systems (Figure 5).

However, crosslinking introduces an intrinsic trade-off. Excessive crosslink density restricts local segmental mobility and disrupts the dynamic rearrangement of hydrogen-bond networks required for efficient proton hopping. Within the transport framework, this corresponds to an increase in the effective activation energy (E_a_) due to limited reorientation of H_2_PO_4_^−^ species, resulting in reduced conductivity despite increased rigidity.

This stiffness–transport trade-off defines a narrow optimal window. Insufficient confinement leads to uncontrolled swelling and rapid plasticization at high ADL, whereas excessive confinement disrupts network continuity and limits proton mobility. Successful crosslinked membranes therefore balance macroscopic stability with local transport dynamics.

Compared to backbone chemical tuning (Section 4.2), which enhances intrinsic cohesive energy density, crosslinking operates at a larger structural scale by introducing artificial constraints. In contrast to topological engineering (Section 4.4), which modifies packing without fixed junctions, crosslinking imposes permanent spatial constraints that can either stabilize or over-constrain the network.

Thus, crosslinking should be viewed not as a universal solution, but as a tunable confinement strategy within the chemo-mechanical framework. When properly optimized, it extends the high-ADL operating regime without crossing the plasticization threshold that governs durability limits.

### 4.4. Topological Confinement Without Permanent Junctions

Topological engineering provides a route to mechanical stabilization based on geometric confinement rather than permanent covalent crosslinks. In contrast to crosslinking, which introduces fixed junction points, branching and star-like architectures modify chain packing and entanglement distribution at the mesoscale, enhancing structural robustness while preserving local segmental mobility.

Hyperbranched PBI systems [42] exemplify this approach, achieving high conductivity alongside substantial mechanical strength. By increasing the density of chain termini and branching points, these architectures enhance entanglement-like confinement, redistribute mechanical stress, and reduce large-scale chain slippage under plasticization. At the same time, the absence of rigid crosslinks allows dynamic reorganization of hydrogen-bond networks, maintaining efficient proton transport.

Star-like architectures [43] further extend this concept by combining branched cores with linear arms. This dual topology provides structural anchoring while preserving extended transport pathways, enabling high resistance to deformation without significantly compromising conductivity. These systems demonstrate that mechanical stabilization can be achieved through architectural design rather than chemical fixation.

From a transport–mechanical perspective, topological engineering promotes a balanced response: conductivity remains elevated while mechanical integrity is preserved. Unlike free-volume expansion strategies (Section 3.5), which enhance conductivity at the expense of structural stability, or highly crosslinked systems (Section 3.2), which may restrict transport through excessive rigidity, branched architectures mitigate plasticization without significantly increasing the effective transport barrier.

Mechanistically, this behavior arises from the ability of topological confinement to increase effective entanglement without disrupting hydrogen-bond continuity. The resulting network remains both percolated and mechanically supported, delaying the onset of creep and acid redistribution under operating conditions.

Nevertheless, topological strategies are not without limitations. Excessive branching can increase synthetic complexity, polydispersity, and microstructural heterogeneity. Moreover, without complementary stabilization mechanisms, control of acid distribution and long-term retention remains challenging.

Overall, topological engineering represents a mesoscale confinement strategy that enhances mechanical resilience while preserving proton transport efficiency. By modifying chain packing and effective entanglement without introducing rigid junctions, it enables access to high-conductivity regimes without prematurely crossing the plasticization threshold.

### 4.5. Free-Volume Expansion and the Mechanical Penalty of Carrier-Density-Driven Design

Free-volume expansion represents an alternative route to increasing proton conductivity in PA–PBI membranes. Instead of relying primarily on stronger structural confinement or higher entanglement density, this strategy enlarges the accessible intermolecular space within the polymer matrix before or during acid incorporation, thereby facilitating higher phosphoric acid uptake and promoting the formation of hydrogen-bonded transport pathways.

In coordination-modified systems [29], this approach increases the acid doping level (ADL) from approximately 7 to 9.5 mol·RU^−1^ and raises conductivity from about 0.068 to 0.126 S·cm^−1^ at 160 °C. These changes are consistent with improved acid accommodation and greater network percolation. However, the conductivity gain is accompanied by a marked mechanical penalty: tensile strength decreases from about 75 MPa to roughly 35–45 MPa, indicating substantial weakening of intermolecular cohesion within the polymer matrix.

Within the transport framework introduced in Section 2, free-volume expansion acts mainly on the carrier-density term rather than on the transport-efficiency term. Increasing free volume enables greater acid uptake and therefore increases the number of available proton-conducting species. However, the corresponding activation energies remain relatively high (∼39–47 kJ·mol^−1^), indicating that proton-transfer efficiency is not substantially improved. In other words, conductivity enhancement in these systems arises primarily from a higher proton population, not from a lower hopping barrier or more efficient proton transfer.

This distinction is important because it defines the main limitation of free-volume-driven design. When conductivity is increased mainly by raising ADL, the membrane becomes more dependent on high acid loading to remain competitive. This, in turn, amplifies plasticization, weakens the polymer network, and increases the fraction of more weakly confined acid species. As a result, these systems move toward a region of the design space where conductivity rises, but resistance to deformation, acid redistribution, and long-term durability decline simultaneously.

Mechanistically, increased free volume reduces packing density and increases local segmental mobility. This facilitates acid incorporation, but it also lowers resistance to creep and deformation under thermal and electrochemical stress. In this sense, free volume should be interpreted as an indirect descriptor of looser packing and enhanced mobility, rather than as a direct measurement of intermolecular or supramolecular distance. The latter can only be specifically estimated when supported by dedicated structural characterization or molecular modeling. Under operating conditions, the combination of looser packing, higher acid uptake, and weaker confinement can favor acid redistribution and accelerate degradation.

Free-volume engineering therefore illustrates the mechanical cost of carrier-density-driven conductivity enhancement. While geometric expansion can improve percolation and acid accommodation, it does not by itself resolve the coupled problems of mechanical weakening and acid mobility. Its practical usefulness thus depends on whether the expanded structure is accompanied by compensating stabilization mechanisms, such as backbone rigidity, controlled crosslinking, or other forms of structural confinement.

Overall, coordination-induced free-volume expansion demonstrates that conductivity gains obtained through increased acid accommodation alone may come at a significant mechanical and retention cost. For this reason, free-volume-based strategies are most promising when integrated with complementary stabilization approaches that limit plasticization and suppress migration toward a high-degradation regime.

### 4.6. From Mechanical Weakening to Electrochemical Failure

The combined evidence across high-molecular-weight systems [18], backbone-modified membranes [29,48], crosslinked networks [31], topological architectures [29], and free-volume systems [29] reveals a consistent structural progression governing durability in PA–PBI membranes:

High ADL → Plasticization → Creep and Swelling

This progression represents a general degradation pathway rather than an architecture-specific phenomenon. Although different strategies modify ADL, activation energy, or network topology, mechanical instability emerges once acid population exceeds the structural tolerance of the polymer matrix.

Plasticization weakens interchain cohesion, increasing segmental mobility. As phosphoric acid accumulates, π–π stacking and hydrogen bonding between polymer chains are progressively disrupted, leading to a higher fraction of mobile acid species. While this enhances proton transport, it simultaneously increases acid diffusivity within the membrane.

Beyond a critical mobility threshold, creep and volumetric swelling become pronounced. Under operating conditions, mechanical stresses, arising from thermal gradients, interfacial heterogeneity, and current-induced expansion, further accelerate structural relaxation. This promotes localized acid redistribution, particularly near catalyst–membrane interfaces.

Local acid depletion reduces proton conductivity and increases ohmic resistance, which manifests as an increase in dV/dt. The transition from structural softening to electrochemical degradation is therefore continuous and causally linked.

Membranes with high tensile strength cluster in the low-decay regime, while mechanically softened systems exhibit accelerated degradation. High-MW membranes exemplify the stable regime, whereas systems with reduced mechanical integrity show decay rates exceeding two orders of magnitude under comparable conditions.

This framework explains why high σ alone cannot ensure durable performance. Once hydrogen-bond network percolation is achieved, further increases in ADL provide diminishing gains in conductivity but significantly increase mechanical vulnerability. The onset of plasticization therefore defines a practical upper bound for stable operation.

Importantly, dV/dt emerges as an integrated durability metric. Unlike σ, which reflects instantaneous transport, dV/dt captures the cumulative effects of creep, acid redistribution, and interfacial degradation, providing a direct link between structural evolution and device performance.

In summary, durability in PA–PBI membranes is governed by the balance between network percolation and structural confinement. The progression from high ADL to plasticization, creep, and electrochemical failure represents a universal degradation pathway. This framework provides the basis for the retention and lifetime analysis presented in Section 5.

### 4.7. Design Implications of Chemo-Mechanical Coupling

The analysis in Section 4.1, Section 4.2, Section 4.3, Section 4.4, Section 4.5 and Section 4.6 demonstrates that plasticization in PA–PBI membranes is not an anomaly, but an intrinsic consequence of acid-mediated proton conduction. Once hydrogen-bond networks reach percolation at ADL ≈ 8–10 mol·RU^−1^, the same structural rearrangements that enable efficient proton transport inevitably weaken cohesive interactions within the polymer matrix.

Effective membrane design must therefore shift from attempting to eliminate plasticization to managing it through multi-scale stabilization. Across the architectural strategies discussed in Section 3, successful systems operate within a narrow structural corridor where high conductivity is achieved without crossing the mechanical instability threshold.

This corridor corresponds approximately to ADL ≈ 10–12 mol·RU^−1^, where σ ≥ 0.14–0.18 S·cm^−1^ can be sustained under anhydrous conditions while mechanical softening remains controllable. Within this window, stabilization must be achieved across multiple length scales.

Backbone tuning increases cohesive energy density and delays chain separation. Molecular weight and branching enhance entanglement and suppress creep. Controlled crosslinking limits macroscopic swelling while preserving local hydrogen-bond dynamics. Topological confinement redistributes stress, and energetic stabilization reduces E_a_, enabling high σ without excessive acid loading.

In the conductivity–mechanical landscape, optimal membranes occupy a narrow diagonal corridor in which conductivity remains high while tensile strength is sufficient to suppress creep and acid redistribution. Systems that move outside this region, either through excessive ADL or insufficient confinement, drift toward the plasticization boundary and exhibit increased voltage decay.

The key implication is that conductivity optimization cannot be pursued independently of mechanical stabilization. Beyond σ ≈ 0.14 S·cm^−1^, further gains yield diminishing returns if structural integrity is compromised. In contrast, modest improvements in mechanical resilience can significantly extend operational lifetime.

Thus, plasticization management emerges as the defining criterion for PA–PBI membrane design. Architectures that balance moderate-to-high ADL with structural and energetic stabilization are most likely to achieve durable performance, whereas systems that exceed the stability corridor without adequate confinement undergo rapid electrochemical degradation.

This perspective provides the structural foundation for Section 5, where acid retention and lifetime are examined quantitatively.

## 5. Acid Retention and Lifetime: From Structural Instability to Electrochemical Failure

If Section 4 established plasticization as the structural origin of instability in PA–PBI membranes, this section examines its electrochemical consequences: acid loss, resistance growth, and long-term voltage decay. While increased ADL enhances conductivity, it simultaneously increases acid mobility, linking structural softening directly to performance degradation under operating conditions.

In PA–PBI membranes, phosphoric acid exists as two dynamically equilibrated populations. A fraction is strongly coordinated to benzimidazole sites, forming relatively stable acid–base complexes that contribute to structural anchoring. The remaining fraction consists of more mobile acid species that form extended hydrogen-bond networks and enable long-range proton transport. As ADL increases, the relative contribution of this mobile fraction grows, enhancing conductivity but also increasing susceptibility to redistribution.

The balance between bound and mobile acid populations therefore governs both transport efficiency and retention stability. At moderate ADL, the network remains percolated while a significant fraction of acid is structurally constrained. At higher ADL, mobile acid species dominate, amplifying redistribution under thermal, mechanical, and electrochemical stress.

Under sustained HT-PEM operation, this balance becomes critical. Thermal gradients, current-induced stress, and mechanical relaxation drive localized acid redistribution, particularly near catalyst–membrane interfaces. Local acid depletion increases ohmic resistance, which in turn accelerates degradation through coupled thermal and electrochemical effects.

Data compiled in Table 6 indicate that acid retention is fundamentally a chemo-mechanical phenomenon. Acid loss is not governed solely by chemical affinity, but by the interplay between mechanical confinement, chain mobility, and structural stability under load. Membranes with higher tensile strength and reduced creep consistently exhibit lower voltage decay rates, whereas mechanically softened systems show accelerated degradation.

Thus, retention cannot be decoupled from plasticization. The electrochemical lifetime of PA–PBI membranes is determined by the extent to which structural design strategies limit acid mobility once percolation is achieved.

The following subsections analyze retention metrics, degradation protocols, and lifetime data within the chemo-mechanical framework established in Section 4.

### 5.1. Metrics and Protocol Dependence in Acid Retention Assessment

Acid retention in PA–PBI membranes is evaluated through multiple experimental approaches, each probing different aspects of acid stability. The most direct metric is the fraction of phosphoric acid remaining after thermal aging, typically determined gravimetrically. Alternatively, retention can be assessed through acid loss under humid or steam exposure, which reflects susceptibility to migration in the presence of water vapor. Indirect metrics are also widely used, including dV/dt under constant current and time-dependent increases in ohmic resistance, both of which capture the cumulative effects of acid redistribution during operation.

However, retention values are strongly dependent on the testing protocol, making cross-comparison between different studies challenging. Anhydrous thermal aging, steam exposure, cyclic operation, and long-term galvanostatic testing impose fundamentally different stresses. As a result, retention percentages alone do not provide a consistent basis for evaluating membrane stability.

This variability is evident when comparing different architectural strategies. Nitrogen-positive engineered membranes [30] exhibit high retention (≈83% after 240 h at 160 °C) under anhydrous conditions [28], reflecting electrostatic stabilization of H_2_PO_4_^−^ species and reduced acid mobility. In contrast, hybrid imi-HPA systems [28], despite achieving high conductivity (σ ≈ 0.1666 S·cm^−1^ at 200 °C), show significant acid loss (~49–54%) under steam exposure, accompanied by pronounced swelling. In these systems, high acid uptake combined with limited structural confinement facilitates acid migration.

These examples highlight that retention must be interpreted in the context of both testing conditions and mechanical stability. Metrics such as dV/dt and resistance growth provide complementary insight, as they integrate the effects of acid redistribution under realistic operating conditions.

A comparative overview of retention behavior and electrochemical durability across representative PA–PBI architectures is summarized in Table 6, including retention values, testing protocols, and voltage decay rates.

The data clearly demonstrate that membranes with comparable conductivity but reduced mechanical stability exhibit significantly higher voltage decay rates. This confirms that durability is governed primarily by structural confinement and acid mobility rather than conductivity alone.

These contrasting results highlight a key principle: acid retention must be interpreted in the context of both testing conditions and mechanical confinement. Anhydrous aging probes the intrinsic stability of acid–polymer interactions, whereas steam exposure introduces additional driving forces for acid migration through hydration gradients and vapor transport. Under galvanostatic operation, electrochemical stress further accelerates localized acid redistribution, particularly at catalyst–membrane interfaces.

Accordingly, retention cannot be reduced to a single percentage value. Instead, it reflects the outcome of coupled chemo-mechanical and electrochemical processes. Meaningful comparison therefore requires simultaneous consideration of ADL, temperature, humidity, aging time, and voltage decay rate, as summarized in Table 6. Acid retention and durability metrics (electrochemical aging).

Within the framework established in Section 2, Section 3 and Section 4, acid retention represents the electrochemical consequence of structural confinement. Membranes with sufficient mechanical integrity and limited acid mobility maintain stable resistance and low dV/dt, whereas systems relying solely on high ADL exhibit rapid degradation under operating conditions.

The following subsection extends this analysis by examining electrochemical aging data and correlating retention behavior with voltage decay and lifetime metrics.

### 5.2. Mechanical Stabilization as a Determinant of Retention Under Electrochemical Load

Mechanical stabilization emerges as a central determinant of acid retention under operating conditions. While Section 4 established that plasticization increases segmental mobility, here this effect is directly linked to electrochemical degradation.

Molecular-weight-controlled systems provide clear quantitative evidence of this coupling. At comparable ADL, membranes with similar conductivity can exhibit dramatically different voltage decay rates depending on mechanical stability. High-MW membranes operate in a low-decay regime, whereas lower-MW analogues exhibit degradation rates differing by orders of magnitude under identical conditions [18]. This disparity confirms that acid redistribution is strongly amplified when mechanical resistance is reduced.

Mechanistically, reduced tensile strength lowers resistance to deformation under thermal and electrochemical stress, enabling localized creep and migration of acid species. This leads to acid depletion near catalyst interfaces, increased interfacial resistance, and accelerated ohmic losses.

Membranes with higher tensile strength cluster in the low-decay region, while mechanically weakened systems exhibit significantly higher dV/dt. The correlation is systematic: as structural confinement decreases, effective acid mobility increases under load.

Data summarized in Table 5 further support this interpretation. Systems with higher tensile strength and controlled plasticization consistently exhibit lower voltage decay rates, whereas mechanically softened membranes show rapid degradation. Acid retention, therefore, cannot be decoupled from the mechanical state of the polymer matrix.

Importantly, retention under electrochemical load is inherently dynamic. Unlike static gravimetric measurements, constant-current operation imposes simultaneous thermal, mechanical, and electrochemical stresses. As a result, dV/dt provides a more integrative metric of retention stability than acid percentage alone.

In summary, mechanical stabilization governs acid retention under load by limiting segmental mobility and suppressing creep-driven acid redistribution. Once a critical mechanical threshold is crossed, even high-conductivity membranes rapidly transition toward electrochemical instability. This reinforces the conclusion that durability in PA–PBI membranes is fundamentally a chemo-mechanical problem.

The following subsection extends this analysis to long-duration behavior and lifetime evolution.

### 5.3. Long-Duration Retention and System-Level Stability

While many studies report short-term conductivity under controlled conditions, relatively few provide long-duration retention and durability data beyond several hundred hours. This gap is critical, as short-term metrics fail to capture the cumulative effects of acid redistribution, mechanical relaxation, and interfacial degradation during sustained high-temperature operation.

Extended electrolyte leaching studies (up to ~1100 h) [49] provide valuable insight into long-term acid dynamics. These results show that retention behavior cannot be reliably extrapolated from short-term experiments: gradual acid redistribution may have limited initial impact on σ but can progressively increase ohmic resistance and accelerate voltage decay beyond a critical threshold.

This highlights the need to integrate retention metrics with time-dependent electrochemical performance. As summarized in Table 3, systems that appear stable over tens of hours may exhibit markedly different behavior over extended operation. Meaningful assessment of durability therefore requires testing under realistic current densities, temperatures, and mechanical conditions.

Even longer operation windows (3000–5000 h) [50] reveal that degradation extends beyond membrane chemistry. Membrane stability becomes strongly coupled to catalyst-layer restructuring, interfacial delamination, and changes in electrode morphology, emphasizing that retention must be evaluated at the membrane–electrode assembly (MEA) level.

At the device scale, localized acid depletion near catalyst interfaces disrupts proton transport continuity and increases contact resistance. As acid redistributes, conduction pathways become non-uniform, leading to nonlinear voltage decay driven by the combined effects of chemical redistribution, mechanical creep, and electrochemical polarization.

Interfacial phenomena further amplify these effects. Mechanical softening can induce dimensional mismatch between membrane and catalyst layers, promoting microcracking and partial delamination. These structural defects accelerate acid migration and exacerbate performance degradation.

Consequently, lifetime prediction in PA–PBI systems cannot rely solely on static retention percentages or initial conductivity. Instead, it requires integration of membrane chemistry, mechanical stability, and MEA architecture. In this context, dV/dt emerges as a practical and integrative metric linking membrane-level processes to device-level durability.

In summary, long-duration studies demonstrate that acid retention must be evaluated under realistic operating conditions and within the complete electrochemical system. The stability corridor identified in Section 4 therefore represents not only a material constraint but a system-level boundary governed by the interplay between polymer structure, acid dynamics, and interfacial behavior.

The following subsection synthesizes these insights into guidelines for predictive lifetime engineering.

### 5.4. Mechanistic Interpretation: Bound vs. Free Acid Populations

A mechanistic understanding of acid retention in PA–PBI membranes requires distinguishing between different populations of phosphoric acid within the polymer matrix. Spectroscopic and structural analyses [51] indicate that acid molecules in doped PBI are not homogeneous in their binding environment. Instead, at least two distinct populations coexist: strongly coordinated acid molecules bound to benzimidazole nitrogen sites and more mobile acid clusters participating in extended hydrogen-bond networks.

The strongly coordinated population forms acid–base complexes that anchor phosphoric acid directly to the polymer backbone. These molecules are relatively constrained and contribute primarily to structural stabilization. By contrast, the more mobile acid fraction forms hydrogen-bonded chains between H_2_PO_4_^−^ and H_3_PO_4_ species. This mobile network is responsible for long-range proton hopping and therefore contributes disproportionately to conductivity.

The coexistence of these two populations introduces an intrinsic trade-off. Increasing ADL enhances the size and connectivity of the hydrogen-bond network, thereby increasing σ. However, it also increases the fraction of loosely bound acid clusters that are susceptible to redistribution under thermal, mechanical, or electrochemical stress.

This interplay becomes evident when comparing distinct architectural strategies. Electronic site engineering [30] strengthens acid–polymer interactions through electrostatic stabilization, effectively increasing the proportion of structurally anchored acid species. In these systems, σ = 0.185 S·cm^−1^ at 180 °C was achieved with low activation energy (13–16 kJ·mol^−1^) and high acid retention (83% after 240 h at 160 °C). The reduced *E_a_* suggests efficient proton hopping, while the enhanced acid–polymer interaction reduces the fraction of highly mobile acid clusters.

In contrast, free-volume expansion via coordination increases ADL and σ but does not reduce activation energy, which remains in the range of 39–47 kJ·mol^−1^ [29]. In such systems, geometric expansion increases the total acid population but does not intrinsically stabilize acid binding. The fraction of mobile acid species rises, increasing the risk of redistribution. Thus, although σ improves, retention stability may not.

This relationship can be conceptualized schematically as:

High ADL → Higher fraction of mobile acid → Increased migration risk

Mechanical confinement (Section 4.1, Section 4.2, Section 4.3 and Section 4.4) and energetic stabilization (Section 2.2) shift this balance. High molecular weight, branching, and moderate crosslinking restrict macroscopic deformation and reduce the extent to which mobile acid clusters can reorganize under stress. Electronic site engineering lowers the energy barrier for proton hopping, enabling high conductivity without excessive mobile acid accumulation.

The transition from localized acid–base complexes to extended hydrogen-bond networks marks the onset of high conductivity. However, once the network becomes dominated by mobile acid species, the system approaches the plasticization boundary further demonstrates that membranes with insufficient confinement exhibit higher voltage decay rates, reflecting the cumulative effect of acid redistribution.

From this perspective, retention stability is governed by the relative proportions of bound and free acid populations and by the degree to which the polymer matrix constrains their mobility. Architectures that maintain a higher fraction of structurally anchored acid while preserving efficient hopping pathways occupy a favorable region of the multidimensional design space.

Thus, acid retention is not merely a function of chemical affinity but of dynamic balance between transport efficiency and structural confinement. Predictive lifetime engineering must therefore consider not only total ADL but also how architectural design shifts the equilibrium between bound and mobile acid species.

### 5.5. Integrating Retention into the Performance Landscape

The data summarized in Table 5 demonstrate that no single architectural strategy simultaneously maximizes proton conductivity, acid retention, and voltage stability. Instead, each design approach shifts membrane performance along different axes of the multidimensional landscape defined throughout this review.

Nitrogen-positive engineered systems exemplify energetic optimization, achieving high conductivity (σ ≈ 0.185 S·cm^−1^ at 180 °C) alongside strong acid retention (≈83% after 240 h at 160 °C) [30]. By lowering activation energy and strengthening acid–polymer interactions, these membranes reduce the fraction of mobile acid species without requiring extreme ADL. However, such approaches often involve increased synthetic complexity.

High-molecular-weight PBI systems represent the mechanically stabilized regime, exhibiting moderate conductivity (σ ≈ 0.14 S·cm^−1^) but exceptionally low voltage decay rates (≈1.5 μV·h^−1^) [18]. In these systems, entanglement-driven confinement suppresses acid redistribution under load.

By contrast, hybrid imi-HPA systems [28] achieve elevated conductivity (σ ≈ 0.1666 S·cm^−1^ at 200 °C) but suffer substantial acid loss (~49–54% under steam exposure). Their high ADL enhances carrier density but increases susceptibility to migration in the absence of sufficient confinement.

When interpreted within the mechanical–decay relationship, these systems occupy distinct regions of the performance landscape. Membranes combining high tensile strength with low dV/dt define a stable operating regime, whereas systems with reduced mechanical integrity and elevated acid mobility drift toward rapid degradation.

This mapping highlights a central conclusion: acid retention is not an independent material property, but the electrochemical consequence of structural design. Conductivity, mechanical stability, and retention form a coupled triad, and optimizing one parameter in isolation inevitably leads to instability.

Accordingly, lifetime engineering in PA–PBI membranes requires balancing multiple interdependent variables within a constrained operating window. In practical terms, durable systems must simultaneously optimize:

• ADL, ensuring sufficient carrier density without excessive mobile acid fraction,
• mechanical confinement, via molecular weight, branching, or controlled crosslinking,
• energetic stabilization, lowering E_a_ without extreme doping,
• swelling suppression, limiting creep and volumetric expansion.

This combination defines a stability corridor in which membranes exhibit low dV/dt, stable resistance, and sustained performance. Architectures that fall outside this region, whether by excessive ADL or insufficient confinement, may achieve high initial σ but fail under long-term operation.

Thus, retention and lifetime are emergent properties of integrated structural design rather than isolated chemical attributes. The following section translates this framework into device-level performance analysis.

### 5.6. Toward Standardized Lifetime Evaluation

The literature on PA–PBI membranes reveals substantial variability in aging protocols and durability metrics. Steam exposure, anhydrous thermal aging, galvanostatic operation, open-circuit voltage (OCV) holds, and potential cycling are often conducted under widely differing conditions of temperature, current density, and environment. As a result, retention percentages and decay rates reported across studies are frequently not directly comparable.

Each protocol probes a distinct aspect of degradation. Steam exposure reflects vapor-driven migration and swelling, anhydrous aging captures thermally activated redistribution, and galvanostatic operation introduces coupled electrochemical and mechanical stresses. Without consistent testing frameworks, quantitative benchmarking across architectures remains inherently limited.

Although Table 7 consolidates available data, clear inconsistencies in reporting persist. Many studies report σ and P_max in detail but lack retention metrics, while others omit voltage decay rates or post-aging mechanical properties. Yet tensile strength and dV/dt are strongly correlated, highlighting the importance of integrating mechanical characterization into durability assessment.

A standardized evaluation framework is therefore essential. At a minimum, studies should report acid retention under defined aging conditions, dV/dt under controlled current density and temperature, and time-resolved resistance evolution to distinguish between bulk and interfacial degradation. Mechanical properties after aging, particularly tensile strength or creep resistance, should also be included to directly link structural softening with performance loss.

Beyond experimental consistency, predictive lifetime assessment requires coupling transport physics with mechanical and diffusion processes. Acid mobility should be quantified through effective diffusion coefficients under load, while creep behavior and interfacial stress must be incorporated into degradation models. Multiscale approaches that integrate membrane properties with MEA-level behavior are particularly critical.

Ultimately, robust lifetime prediction will depend on harmonized protocols and integrated mechanistic modeling. The goal is to define quantitative stability criteria that extend beyond isolated conductivity values and instead capture the structural and energetic conditions required for sustained operation.

With this foundation, the following section examines device-level performance across architectures, translating chemo-mechanical and retention principles into practical performance limits.

## 6. Device-Level Implications and Performance Benchmarking

While Section 2, Section 3, Section 4 and Section 5 established the physicochemical principles governing transport, plasticization, and acid retention in PA–PBI membranes, their ultimate validation lies in device-level performance under realistic HT-PEM conditions. Ex situ conductivity, although essential for mechanistic insight, does not directly translate into power output or durability unless mechanical stability, acid retention, and interfacial integrity are maintained during operation.

In practical membrane–electrode assemblies (MEAs), membranes are subjected simultaneously to elevated temperature (150–180 °C), electrochemical polarization, mechanical compression, and spatial gradients in acid concentration. Under these conditions, the chemo-mechanical constraints identified in Section 3, Section 4 and Section 5 become operationally decisive. Materials that exhibit high σ under controlled conditions may fail in devices if plasticization promotes acid redistribution at the membrane–electrode interface.

This section integrates single-cell performance metrics with structural considerations to define the practical performance envelope of PA–PBI membranes.

### 6.1. Conductivity Versus Power Density: Non-Linear Scaling

A linear relationship between σ and P_max is often assumed, as lower membrane resistance should reduce ohmic losses. However, the literature data show that this relationship is strongly non-linear and architecture-dependent.

High-molecular-weight PBI membranes provide a clear example. Despite moderate conductivity (σ ≈ 0.14 S·cm^−1^ at 160 °C), these systems achieve P_max ≈ 295 mW·cm^−2^ under H_2_/air conditions, supported by excellent mechanical stability and low voltage decay [18]. This demonstrates that structural robustness can compensate for moderate σ at the device level.

In contrast, nitrogen-positive engineered membranes [30] combine high conductivity (σ ≈ 0.185 S·cm^−1^) with low activation energy, achieving P_max ≈ 550 mW·cm^−2^ under H_2_/O_2_ conditions. Here, both transport efficiency and favorable kinetics contribute to improved performance.

Hybrid imi-HPA systems [28] further highlight the complexity of this relationship. Although these membranes exhibit comparable conductivity (σ ≈ 0.1666 S·cm^−1^), their peak power density is lower (P_max ≈ 454 mW·cm^−2^), and retention is significantly reduced. This indicates that high initial conductivity does not guarantee sustained device performance when structural confinement is insufficient.

These examples demonstrate that device performance emerges from a multidimensional balance between conductivity, mechanical stability, and acid retention. Table 4 summarizes representative single-cell performance data across different architectures.

Several additional factors further modulate P_max, including catalyst loading, gas composition (H_2_/air vs. H_2_/O_2_), operating temperature, membrane thickness, and interfacial resistance within the MEA. For example, operation under H_2_/O_2_ generally yields higher P_max due to reduced mass-transport limitations, while increased temperature enhances electrode kinetics and partially compensates for moderate σ.

Thus, while high conductivity is necessary, it is not sufficient for high device performance. The effective resistance under operating conditions depends on the interplay between transport, structural stability, and interfacial behavior. Membranes optimized solely for σ may exhibit high initial power output but suffer from rapid degradation.

In summary, device-level benchmarking confirms that the conductivity–power relationship is inherently non-linear and architecture-dependent. Performance must therefore be interpreted within the broader chemo-mechanical framework established in Section 4 and Section 5. The following subsection examines how durability and voltage decay further constrain the operational envelope of PA–PBI membranes.

### 6.2. Mechanical Stability and Voltage Decay Under Load

Device-level performance in HT-PEM systems must be evaluated over operational time rather than solely through initial polarization curves. High peak power density is insufficient if structural degradation leads to rapid increases in internal resistance. For this reason, dV/dt under constant-current operation emerges as a critical benchmarking parameter.

Comparative studies clearly demonstrate that membranes with similar conductivity can exhibit dramatically different degradation rates depending on mechanical stability. High-MW systems operate in a low-decay regime, whereas lower-MW analogues show degradation rates differing by orders of magnitude under identical conditions [18]. This disparity confirms that resistance growth under load is governed primarily by structural confinement rather than by σ alone.

Mechanistically, plasticization reduces tensile strength and increases segmental mobility, lowering resistance to deformation under thermal and electrochemical stress. This enables localized creep and acid redistribution, particularly near membrane–electrode interfaces. The resulting acid depletion increases interfacial resistance and accelerates ohmic losses, which manifest as increased dV/dt.

Membranes with higher tensile strength cluster in the low-decay region, while mechanically weakened systems exhibit significantly higher degradation rates. The trend is systematic, indicating a direct causal link between structural softening and electrochemical instability.

The importance of confinement is further highlighted by hybrid systems. Although some architectures achieve high σ and P_max, their limited mechanical stability leads to significant acid loss and accelerated degradation under operating conditions. This demonstrates that high initial performance does not guarantee long-term stability in the absence of sufficient structural support.

Voltage decay therefore integrates multiple degradation processes, including acid mobility, mechanical creep, interfacial degradation, and catalyst-layer restructuring. As such, membranes that exhibit high P_max but elevated dV/dt are unlikely to be viable for practical applications.

For meaningful benchmarking, dV/dt must be evaluated alongside σ and P_max. Only by combining instantaneous performance metrics with time-dependent degradation data can the true operational envelope of PA–PBI membranes be defined.

The following subsection integrates these observations into a multidimensional performance envelope that couples conductivity, power density, and durability across architectures.

### 6.3. Operating Temperature and Catalyst Layer Effects

Operating temperature exerts a dual influence on HT-PEM device performance. On one hand, conductivity increases with temperature following Arrhenius behavior; on the other, elevated temperature enhances electrode kinetics, reducing activation losses at both electrodes.

POSS-assisted crosslinked membranes [47] illustrate this coupling. Despite moderate conductivity (σ ≈ 0.097 S·cm^−1^ at 180 °C), these systems achieve high power density (P_max ≈ 461 mW·cm^−2^ at 200 °C), as elevated temperature compensates for limited intrinsic transport by accelerating reaction kinetics and reducing polarization losses.

This highlights a key point: σ and P_max cannot be interpreted independently of operating temperature. Membranes with moderate conductivity may deliver competitive performance at higher temperatures, provided mechanical stability and retention are maintained. Conversely, high σ alone does not guarantee high P_max if temperature is limited or interfacial losses dominate.

Beyond bulk membrane properties, the catalyst layer (CL) plays a decisive role. Bipyridine-functionalized PBI ionomers [52] have been shown to enhance power density by improving proton conduction within the catalyst layer and optimizing acid distribution at the electrode–membrane interface. This demonstrates that effective proton transport in the MEA depends not only on membrane conductivity, but also on ionomer functionality and interfacial architecture.

From a structural perspective, the MEA introduces additional constraints. Mechanical compression, differential swelling between membrane and catalyst layer, and local acid redistribution under load can all modify effective transport pathways. As a result, ex situ conductivity does not directly reflect in situ performance.

Optimal performance is achieved when transport, mechanical confinement, and interfacial proton conduction are simultaneously balanced.

Thus, membrane performance cannot be evaluated in isolation. A membrane optimized for bulk conductivity may underperform if paired with an unsuitable catalyst-layer ionomer, whereas moderate σ combined with optimized MEA design and elevated temperature operation can yield competitive performance.

In summary, device-level behavior in HT-PEM systems emerges from the interplay of membrane transport, electrode kinetics, and interfacial architecture. Meaningful benchmarking must therefore integrate material properties with MEA-level design considerations.

### 6.4. Defining the HT-PEM Performance Envelope

Across the literature examined in Section 3, Section 4 and Section 5, a practical HT-PEM performance envelope can be identified by jointly considering proton conductivity, peak power density, and voltage decay rate under realistic operating conditions. Although individual studies may report values outside these ranges, most stable PA–PBI systems cluster within a region defined by:

- 0.10 ≲ σ ≲ 0.20 S·cm^−1^
- 300 ≲ P_max ≲ 600 mW·cm^−2^
- 1 ≲ dV/dt ≲ 100 μV·h^−1^

These ranges represent experimentally accessible performance boundaries under anhydrous or low-humidity conditions, rather than intrinsic or universal limits. Importantly, this region should be interpreted as a multivariate stability corridor emerging from the combined behavior of transport, mechanical, and retention properties, rather than as a single-variable conductivity threshold.

Membranes with σ > 0.18 S·cm^−1^ can achieve high initial power densities, particularly under optimized operating conditions [30]. However, as shown in Section 4 and Section 5, such performance is often obtained at the expense of mechanical stability and acid retention. Without sufficient structural confinement or energetic stabilization, these systems exhibit increased acid mobility, leading to higher voltage decay rates and reduced long-term durability.

High-molecular-weight PBI membranes define a contrasting regime, characterized by moderate conductivity (σ ≈ 0.14 S·cm^−1^), moderate power density (P_max ≈ 300 mW·cm^−2^), and exceptionally low decay rates (dV/dt ≈ 1–2 μV·h^−1^) [18]. These systems represent a mechanically stabilized baseline within the performance envelope, demonstrating that durability can compensate for moderate transport performance.

Nitrogen-positive engineered membranes approach the upper boundary of the conductivity–power corridor by combining high σ and P_max with relatively strong retention, enabled by reduced activation energy and improved acid–polymer interactions. In contrast, hybrid and free-volume-modified systems achieve high σ primarily through increased ADL or acid accommodation, but often drift toward higher degradation rates due to insufficient mechanical confinement and increased acid mobility.

Taken together, these trends show that optimal performance does not coincide with maximum conductivity. Instead, it emerges at the intersection of sufficient σ, adequate mechanical stability, and low voltage decay rate. Notably, similar conductivity values may correspond to markedly different durability regimes depending on the degree of plasticization and structural confinement.

From a design perspective, the performance envelope emphasizes balance rather than extremes. Conductivity below ~0.10 S·cm^−1^ limits current density, while values above ~0.20 S·cm^−1^ are often associated with increased plasticization and structural instability unless counterbalanced by stabilization strategies. Thus, the apparent “conductivity corridor” reflects the intersection between hydrogen-bond network percolation and the onset of chemo-mechanical degradation, rather than a purely transport-defined limit.

The HT-PEM performance envelope therefore defines a constrained region in which transport, mechanical integrity, and durability are simultaneously satisfied. Architectures operating within this corridor can deliver sustained performance, whereas those optimized for a single parameter, particularly σ, tend to suffer from accelerated degradation.

From this perspective, the stability corridor provides a conceptual bridge toward lifetime prediction: membranes operating within this region exhibit sufficiently low dV/dt to sustain operation over extended timescales, whereas deviations, either toward insufficient conductivity or excessive ADL-driven plasticization, lead to rapid performance decay.

The following section synthesizes these insights into quantitative design rules for next-generation PA–PBI membranes.

### 6.5. Implications for Design Optimization

Device-level benchmarking across PA–PBI architectures demonstrates that maximizing proton conductivity alone is insufficient to ensure durable high-temperature operation. While σ is necessary to achieve competitive current density and power output, long-term performance is governed by the coupled interplay between transport efficiency, mechanical stability, and acid retention.

The performance envelope defined in Section 6.4 indicates that practical HT-PEM membranes must operate within a constrained stability corridor, where conductivity is sufficiently high to sustain proton transport, yet mechanical integrity and acid confinement remain robust. Membranes that exhibit stable operation under anhydrous conditions typically combine σ ≈ 0.14–0.18 S·cm^−1^ with low voltage decay rates (dV/dt ≲ 5 μV·h^−1^) and sufficient mechanical strength to suppress creep at operating temperature.

Within this framework, conductivity represents a necessary but non-sufficient condition. Increasing σ through high ADL or free-volume expansion can enhance initial performance, but often at the expense of mechanical stability. Once plasticization exceeds a critical threshold, acid redistribution accelerates and durability rapidly declines.

Mechanical confinement therefore becomes a central design requirement. Strategies such as high molecular weight, branching, controlled crosslinking, and backbone rigidification limit segmental mobility and suppress creep, stabilizing the membrane under operating conditions. Without adequate confinement, even highly conductive systems exhibit rapid degradation.

Acid retention reflects the electrochemical consequence of this structural stability. Architectures that limit the fraction of mobile acid species, through energetic stabilization or controlled topology, are more likely to maintain low dV/dt and stable resistance over time.

Across the architectures examined, the most effective systems combine moderate ADL (≈10–12 mol·RU^−1^) to ensure network percolation, sufficient structural confinement to delay plasticization, and reduced activation energy to enhance transport efficiency without excessive acid loading.

Thus, device-level optimization in PA–PBI membranes requires balancing coupled variables rather than maximizing individual metrics. The interplay between conductivity, mechanical stability, and retention defines the practical operating window for durable HT-PEM performance.

## 7. Integrated Design Rules and Outlook

The preceding sections demonstrate that phosphoric acid-doped PBI membranes do not follow a simple “higher conductivity is better” paradigm. While early efforts focused on increasing σ through higher acid doping or enhanced hydrogen-bond networks, it is now clear that conductivity, mechanical stability, and acid retention are intrinsically coupled.

A consistent theme emerges: transport enhancement and structural destabilization are fundamentally linked. Increasing ADL improves network connectivity and proton flux but weakens cohesive interactions within the polymer matrix. Lowering activation energy enhances hopping efficiency, yet without sufficient confinement, mobile acid species remain prone to redistribution. Similarly, free-volume expansion increases carrier density but often accelerates plasticization and long-term degradation.

As a result, PA–PBI membranes must be understood within a multidimensional performance landscape in which transport energetics, polymer architecture, mechanical integrity, and retention stability are interdependent. Device-level analysis confirms that peak performance and durability are determined not by isolated conductivity values, but by the coordinated optimization of these variables.

The objective of this section is to synthesize the mechanistic and quantitative insights developed in Section 2, Section 3, Section 4, Section 5 and Section 6 into integrated design rules for next-generation PA–PBI membranes. Rather than proposing isolated modifications, we define a stability corridor within which high conductivity can be sustained over technologically relevant timescales.

By integrating transport physics, chemo-mechanical coupling, retention behavior, and device-level performance, this section outlines the key design principles required to transition PA–PBI membranes from laboratory-optimized materials to predictive, durable electrolytes for high-temperature fuel cell applications.

### 7.1. A Multidimensional Design Space

The analysis developed throughout this review supports a unified view in which PA–PBI membranes must be interpreted within a multidimensional design space rather than along a single conductivity axis. Proton transport, mechanical stability, and lifetime performance are governed by coupled variables operating across molecular, mesoscale, and device levels.

At the membrane level, conductivity can be expressed conceptually as:

σ ∝ f(ADL, E_a_, network topology)

where ADL governs proton carrier density and hydrogen-bond network percolation, E_a_ controls proton hopping efficiency, and network topology, defined by molecular weight, crosslinking, branching, and backbone structure, determines both pathway continuity and mechanical confinement.

However, transport alone does not define performance. Electrochemical stability introduces an additional level of coupling:

dV/dt ∝ f(acid mobility, mechanical stability, structural evolution)

In this formulation, acid mobility reflects the fraction of loosely bound species capable of redistribution, while mechanical stability constrains creep and plasticization. As shown in Section 4, mechanical properties can be described semi-empirically as:

S = S_0_ exp(−α·ADL)

linking acid content directly to structural weakening and, indirectly, to degradation rate.

Together, these relationships define a multidimensional design space governed by four principal variables:

· Proton conductivity (σ), representing instantaneous transport efficiency
· Mechanical strength, constraining plasticization and creep
· Acid retention and mobility, governing redistribution under load
· Voltage decay rate (dV/dt), integrating degradation processes at the device level.

Within this framework, optimal membranes occupy a constrained region in which conductivity is sufficiently high (typically ≥ 0.14–0.18 S·cm^−1^ under anhydrous conditions), while mechanical integrity and retention stability suppress rapid degradation. Systems that maximize σ without adequate confinement migrate toward regions of elevated dV/dt, whereas overly rigid systems may remain stable but underperform due to limited proton mobility.

Importantly, this region corresponds to the multivariate stability corridor identified in Section 6.4 and further formalized in the design rules of Section 7.2. This corridor should not be interpreted as a fixed conductivity threshold, but rather as a projection of a higher-dimensional space in which transport, mechanical, and retention constraints intersect.

This perspective shifts the focus from maximizing individual metrics to identifying stability corridors defined by coupled transport, mechanical, and durability constraints. In this sense, predictive membrane design does not rely on a single governing equation, but on positioning materials within this multidimensional space.

Recent studies have also explored advanced diagnostic and control strategies to address voltage degradation at the system level. For example, Tang et al. [53] developed a temperature-sensitive adaptive model enabling real-time monitoring and mitigation of voltage decay, particularly under high-temperature operating conditions. Such approaches provide a complementary framework for durability assessment and highlight the importance of integrating material-level design with system-level monitoring strategies.

The following subsections translate this conceptual framework into quantitative design rules and practical guidelines for next-generation PA–PBI membranes.

### 7.2. Quantitative Design Rules

The comparative analysis of high-molecular-weight systems [18], electronically engineered membranes [30], hybrid architectures [28], free-volume systems [29], and crosslinked networks [31] enables the extraction of quantitative design rules grounded in experimental evidence rather than isolated performance records.

These rules do not prescribe a single material formulation; instead, they define stability corridors within a multidimensional design space, in which transport, mechanical integrity, and durability constraints are simultaneously satisfied.

Rule I: Operate Within the Percolation–Stability Window 

Conductivity increases sharply as ADL approaches ~10 mol·RU^−1^ in PBI systems [18]. At ADL ≈ 10–11 mol·RU^−1^, σ values of ~0.10–0.14 S·cm^−1^ at 160 °C are typically achieved under anhydrous conditions.

However, further increases in ADL do not proportionally enhance σ and instead promote plasticization, swelling, and acid redistribution. The practical target is therefore a percolation–stability window at moderate-to-high ADL (≈10–12 mol·RU^−1^), sufficient to ensure hydrogen-bond network continuity while limiting the fraction of mobile acid species.

This window corresponds to the lower boundary of the multivariate stability corridor identified in Section 6.4, where transport efficiency and structural stability remain balanced.

Rule II: Prioritize Energetic Stabilization over Carrier Density

Electronic site engineering [30] demonstrates that high conductivity (σ ≈ 0.185 S·cm^−1^ at 180 °C) can be achieved with low activation energy (E_a_ ≈ 13–16 kJ·mol^−1^) without extreme ADL.

In contrast, free-volume expansion increases ADL but maintains higher E_a_ (≈39–47 kJ·mol^−1^) [29], requiring larger acid populations to achieve comparable σ. This highlights that reducing E_a_ is a more efficient and structurally stable pathway to conductivity enhancement than increasing carrier density alone, as it enables high σ without shifting the system toward the plasticization boundary

Rule III: Mechanical Stability Sets the Upper Bound for Performance

Durability is highly sensitive to mechanical integrity. At similar ADL, membranes with comparable σ can exhibit degradation rates differing by orders of magnitude [18].

Once tensile strength falls below a critical threshold, creep and acid redistribution accelerate, leading to increased ohmic resistance and voltage decay.

This behavior demonstrates that mechanical stability defines the upper boundary of the stability corridor, beyond which increased conductivity cannot compensate for accelerated degradation.

Rule IV: Carrier-Density Strategies Require Confinement Coupling

Hybrid and free-volume-based approaches can achieve high σ through increased acid uptake but often suffer from reduced mechanical stability and retention.

These strategies must be coupled with structural confinement (e.g., crosslinking, branching, or backbone rigidity) to limit acid mobility. Without such coupling, enhanced carrier density shifts the system outside the stability corridor, leading to accelerated degradation and loss of performance.

Rule V: Device-Level Metrics Are Essential for Benchmarking

High conductivity alone is insufficient for evaluating membrane performance. Meaningful benchmarking requires simultaneous reporting of:

• P_max,
• dV/dt,
• acid retention,
• operating conditions (temperature, current density, humidity).

Extended durability studies (>1000 h) remain limited but are critical for realistic assessment. Device-level stability must therefore be treated as an essential performance criterion rather than a secondary metric. In this context, dV/dt acts as an integrative descriptor linking transport, mechanical properties, and acid mobility, and provides a direct measure of whether a given membrane operates within the stability corridor.

Collectively, these design rules define a stability corridor in which conductivity, mechanical robustness, and retention stability are simultaneously satisfied. Importantly, this corridor should be interpreted not as a fixed conductivity target, but as a projection of a higher-dimensional design space, in which ADL, activation energy, network topology, and mechanical confinement jointly determine performance and durability.

Membranes operating within this region can achieve sustained HT-PEM performance, whereas architectures optimized for a single parameter tend to drift toward rapid degradation.

The following subsection outlines future directions for advancing PA–PBI membrane design.

### 7.3. Toward Predictive Lifetime Engineering

Despite significant advances in membrane chemistry and acid-retention strategies, the field of PA–PBI-based HT-PEMFCs still lacks a fully mature framework for predictive lifetime engineering. One of the main limitations is the absence of harmonized durability and aging protocols. In the literature, membrane stability and lifetime are assessed under highly diverse conditions, including constant-current operation, temperature variation, phosphoric acid loss measurements, start–stop operation, and post-mortem analyses of MEAs. Because these protocols probe different degradation pathways, direct comparison across studies remains difficult, and the predictive value of isolated durability data is inherently limited.

A second limitation is that lifetime is often inferred from voltage decay alone, without a consistent mechanistic decomposition of the underlying causes. Available studies show that voltage degradation in HT-PEMFCs cannot be assigned to a single factor. Instead, it reflects the coupled evolution of activation losses, ohmic resistance, phosphoric-acid redistribution or evaporation, and catalyst/electrode degradation. Kim et al. developed a one-dimensional hybrid degradation model in which lifetime was predicted from the time evolution of activation and ohmic overpotentials under different current densities and PA doping levels [54]. Their analysis showed that reduced PA doping level strongly accelerates degradation by increasing ohmic and charge-transfer resistance, while current density has a weaker effect on degradation rate under the modeled conditions.

Subsequent work moved from simple hybrid lifetime extrapolation toward more explicit overpotential-resolved degradation modeling. Kang et al. proposed a semi-empirical framework in which activation, ohmic, and concentration overpotentials evolve dynamically with time and temperature, allowing both initial performance and long-term degradation to be simulated within a unified model [55]. Their results indicate that the dominant degradation contribution at elevated temperature arises from activation overpotential, while PA doping level and inlet pressure mainly influence lifetime through their effect on conductivity and transport losses. Importantly, this study also showed that higher temperature may slightly improve initial performance but substantially shorten predicted lifetime, emphasizing that durability optimization cannot be reduced to conductivity maximization alone.

A further advance was achieved when degradation mechanisms were separated more explicitly in multidimensional simulations. Won et al. used a three-dimensional degradation model to distinguish the relative roles of PA loss and electrochemical surface area (ECSA) reduction during constant-current operation [56]. Their analysis showed that if voltage decay is attributed to PA loss alone, the implied PA-loss rate becomes unrealistically high compared with experimental measurements. Better agreement with degradation data was obtained only when both PA loss and ECSA reduction were considered simultaneously, leading to the important conclusion that catalyst/electrode aging remains a major contributor even under nominally steady operating conditions. This is a key point for the field, because it shows that lifetime prediction must move beyond membrane-only metrics and account for coupled membrane–electrode degradation.

Taken together, these studies show that current lifetime-prediction approaches for PA–PBI systems can be grouped into three broad classes: hybrid one-dimensional degradation models, semi-empirical overpotential-based models, and multidimensional mechanism-separation models, as summarized in Table 8. These approaches differ in complexity, required inputs, and physical resolution, but all share a common feature: they remain strongly dependent on fitted degradation parameters and literature-specific operating windows. These approaches differ in complexity, required inputs, and physical resolution, but all share a common feature: they remain strongly dependent on fitted degradation parameters and literature-specific operating windows. In other words, the field is progressing toward predictive lifetime engineering, but present models are still better described as semi-predictive than fully generalizable.

Within this perspective, the chemo-mechanical coupling described in Section 4 provides a direct link between material properties and lifetime evolution. In particular, the semi-empirical relation:

$$S=S_{0}exp(-\alpha \cdot ADL)$$

can be interpreted as a structural constraint on durability. As tensile strength decreases with increasing ADL, the susceptibility to creep and acid redistribution increases, which in turn accelerates voltage decay.

In a simplified form, this coupling can be expressed as:

$$\frac{dV}{dt}\propto f(S^{-1},\;ADL)$$

indicating that membranes with lower mechanical strength exhibit higher degradation rates under comparable operating conditions.

Although such relations remain phenomenological, they highlight that lifetime in PA–PBI systems is not governed by conductivity alone, but by the coupled evolution of mechanical integrity and acid mobility. This provides a conceptual bridge between structure–property relationships and predictive lifetime modeling.

To make lifetime prediction more transferable, future studies should combine standardized experimental protocols with model structures that explicitly connect measurable variables to degradation-state evolution. At a minimum, durability studies should report acid doping level, operating temperature, current density, voltage-decay rate, and time-resolved resistance evolution using directly comparable protocols. Post-aging characterization should also include membrane thickness, residual acid content, and, whenever possible, catalyst-surface-area loss and mechanical-property changes. These quantities provide the bridge between phenomenological voltage decay and physically interpretable degradation variables.

From a modeling perspective, the next step is not simply to build more complex simulations, but to construct hierarchical prognostic frameworks. In such a framework, simpler voltage-decay or semi-empirical models could be used for rapid screening, while higher-fidelity models would be used to distinguish the relative contributions of PA loss, conductivity decay, and ECSA reduction. Ultimately, predictive lifetime engineering for PA–PBI membranes will require models that link transport, acid retention, catalyst aging, and structural evolution into a unified description of performance decay over time.

Thus, the future of PA–PBI membrane development lies not only in achieving higher proton conductivity, but in predicting how long a given membrane–MEA architecture can remain inside a defined stability corridor under realistic operating conditions. Reaching this stage would shift the field from descriptive durability studies to true durability forecasting, thereby helping bridge the gap between laboratory optimization and practical long-term deployment.

**Table 8:** Representative approaches for lifetime prediction in PA–PBI-based HT-PEMFCs.

Comparison Criterion	Hybrid 1D Degradation Model	Semi-Empirical Overpotential-Based Model	3D Mechanism-Separation Model
Representative study	Kim et al.	Kang et al.	Won et al.
Source	[54]	[55]	[56]
Model type	Hybrid physics + empirical 1D model	Semi-empirical degradation-rate model	Multidimensional numerical degradation model
Main purpose	Predict lifetime under different current densities and PA doping levels	Predict performance decay and lifetime under varying operating conditions	Separate and compare degradation contributions from PA loss and ECSA reduction
Core lifetime variable	Cell-voltage decay to end-of-life threshold	Time evolution of activation, ohmic, and concentration overpotentials	Voltage degradation reproduced through coupled PA-loss and ECSA-decay assumptions
Main degradation inputs	PA doping level, current density, temperature	Reference exchange current density, PA doping level, oxygen molar fraction, temperature	PA loss rate, PA doping level, ECSA decay, local current distribution
Mechanisms explicitly represented	Activation and ohmic losses	Activation, ohmic, and concentration losses	PA loss and catalyst-surface-area loss
Strength	Interpretable and computationally simple; useful for condition screening	Captures trade-offs between initial performance and lifetime	Mechanistically richer; clarifies dominant degradation pathways
Main limitation	Strong dependence on empirical assumptions and simplified geometry	Limited transferability across MEA designs and aging histories	Higher complexity and continued dependence on fitted degradation parameters
Main conclusion for the field	Low PA doping level strongly shortens predicted lifetime	Temperature strongly affects lifetime, mainly through activation losses	PA loss alone is insufficient to explain voltage decay; ECSA loss remains major

### 7.4. Outlook: The Next Generation of PA–PBI Membranes

The next generation of PA–PBI membranes must move beyond incremental optimization and target a clearly defined stability corridor in which high proton conductivity, mechanical robustness, and long-term durability coexist. The quantitative analysis presented in this review indicates that competitive HT-PEM membranes should aim to simultaneously achieve σ ≳ 0.20 S·cm^−1^ under anhydrous conditions, tensile strength sufficient to suppress creep at operating temperature (≈≥20 MPa), voltage decay rates below ~5 μV·h^−1^ under standardized load, and acid retention above ~90% after extended aging.

These targets are ambitious but grounded in the upper performance ranges reported across the literature. The key challenge is achieving them simultaneously within a single architecture. As demonstrated throughout this review, optimizing one parameter in isolation inevitably shifts the system along another axis, often toward instability.

Reaching these performance levels therefore requires integrated design strategies. Moderate ADL values (≈10–12 mol·RU^−1^) are sufficient to sustain network percolation without exceeding the plasticization threshold. Energetic stabilization of proton hopping sites provides a pathway to enhance σ without excessive acid loading. Structural confinement, through molecular weight, controlled crosslinking, or topological design, suppresses creep while preserving hydrogen-bond dynamics. At the same time, swelling must be minimized to limit acid redistribution under operating conditions.

Future research should focus on mapping the multidimensional performance landscape rather than pursuing isolated record conductivity values. Systematic variation of ADL, activation energy, topology, and confinement under standardized durability protocols and dynamic operating conditions will enable identification of stable operating regions. While direct device-level validation (e.g., MEA peel strength measurements or accelerated stress testing) is essential to quantify interfacial degradation, the present review focuses on identifying the key degradation drivers and proposes a conceptual framework for durability assessment under realistic operating conditions. In this context, dynamic ADL evolution, thermo-mechanical coupling, and acid redistribution are highlighted as critical variables for defining standardized durability protocols and unified failure criteria. Coupled multiscale models integrating acid diffusion, mechanical creep, and electrochemical degradation will be essential for transitioning from empirical optimization to predictive design.

A further direction for the next generation of PA–PBI membranes is the integration of artificial intelligence (AI) and machine learning (ML) into membrane design and optimization workflows. Recent reviews on ion-conducting membranes and PEM-based electrochemical systems indicate that ML can already support materials discovery, property prediction, process optimization, and degradation analysis by identifying non-obvious correlations between composition, microstructure, and performance descriptors [57,58]. For PA–PBI membranes, these approaches are especially attractive because the design space is intrinsically multidimensional, involving acid doping level, polymer architecture, crosslink density, free volume, local hydrogen-bond topology, mechanical reinforcement, and acid-retention behavior.

In practical terms, supervised learning models could be used to predict conductivity, activation energy, acid uptake, or post-aging mechanical properties from experimentally accessible descriptors, while unsupervised learning could help classify membrane families according to shared transport–stability patterns. Bayesian optimization and Gaussian-process-based methods are particularly promising for guiding synthesis campaigns under limited data conditions, whereas graph neural networks may become useful for linking repeat-unit chemistry and local structural motifs to emergent transport properties. Transfer learning may also be relevant for PA–PBI systems, since knowledge extracted from broader proton-conducting membrane datasets could be adapted to smaller PA–PBI-specific datasets.

At the same time, the present review of the field suggests that the main limitation is not algorithm availability, but data quality and standardization. Recent AI-focused reviews emphasize that robust model development still depends on large, consistent, and well-annotated datasets, as well as on reproducible protocols for membrane preparation, testing, and durability assessment. In the specific case of PA–PBI membranes, this means that future studies should report harmonized descriptors spanning composition, acid doping level, conductivity, activation energy, retention, and post-aging structural or mechanical changes. Without this level of data consistency, AI models risk remaining purely correlative and poorly transferable.

Therefore, the most realistic near-term role of AI in PA–PBI development is not autonomous membrane discovery, but decision support within a physics-guided design loop. In such a framework, ML models would accelerate candidate screening, identify promising regions of the design space, and help prioritize experiments, while mechanistic understanding would still be anchored in proton transport physics, acid-network topology, and chemo-mechanical stability. In the longer term, the convergence of standardized datasets, interpretable AI, and multiscale modeling may enable true inverse design of PA–PBI membranes targeted not only for high conductivity, but also for operation inside a defined durability corridor.

Ultimately, progress in PA–PBI membranes depends on shifting from performance maximization to stability-oriented engineering within defined structural limits. By integrating transport physics, molecular architecture, mechanical stabilization, and device-level behavior, these systems can evolve from laboratory materials to robust, predictive electrolytes capable of sustained high-temperature operation over thousands of hours.

Such integration is essential for enabling reliable and scalable HT-PEM fuel cell technologies in hydrogen-based energy systems.

## 8. Conclusions

This review demonstrates that phosphoric acid-doped polybenzimidazole (PA–PBI) membranes cannot be optimized through proton conductivity alone, as transport, mechanical stability, and acid retention are intrinsically coupled.

A unified framework has been established in which membrane performance is governed by a multidimensional design space defined by acid doping level (ADL), activation energy (E_a_), hydrogen-bond network topology, and mechanical confinement. Within this framework, conductivity arises from the interplay between carrier density and proton hopping efficiency, while mechanical stability decreases with increasing ADL due to acid-induced plasticization.

Comparative analysis of molecular architectures shows that different design strategies—molecular weight control, crosslinking, backbone modification, topological engineering, and free-volume tuning—do not independently optimize performance, but instead shift membranes within a coupled transport–mechanical–retention landscape. Device-level data further confirm that similar conductivity values can correspond to markedly different degradation rates, demonstrating that durability is governed primarily by structural confinement and acid mobility rather than by σ alone.

A multivariate stability corridor has been identified, within which PA–PBI membranes achieve σ ≈ 0.14–0.20 S·cm^−1^ while maintaining low voltage decay rates under realistic HT-PEM operating conditions. This corridor reflects the balance between hydrogen-bond network percolation and the onset of chemo-mechanical degradation, rather than a strict conductivity threshold.

Based on this analysis, quantitative design rules emerge: (i) operate within a percolation–stability window at moderate ADL, (ii) prioritize energetic stabilization of proton hopping over excessive carrier density, (iii) ensure sufficient mechanical confinement to suppress creep and acid redistribution, and (iv) evaluate performance using integrated device-level metrics, including dV/dt.

Finally, the transition from empirical optimization to predictive lifetime engineering requires standardized durability protocols and the development of multiscale models linking transport, mechanics, and acid dynamics. In this context, PA–PBI membranes should be designed not to maximize individual properties, but to operate within a constrained multidimensional stability region that ensures sustained performance over technologically relevant timescales.

## Figures and Tables

**Table 1:** Comparison between PFSA and PA–PBI membranes in terms of polymer structure, proton-conducting phase, transport mechanism, humidity dependence, operating temperature window, and key design trade-offs.

Feature	PFSA Membranes	PA–PBI Membranes
Polymer chemistry	Perfluorosulfonic acid polymers with fluorinated backbone and sulfonated side chains	Polybenzimidazole doped with phosphoric acid
Main proton-conducting medium	Water-rich ionic domains	Phosphoric acid hydrogen-bonded network confined in PBI matrix
Proton transport mechanism	Vehicular diffusion and Grotthuss hopping in hydrated channels	Acid-mediated proton hopping through H_3_PO_4_/H-bonded network, assisted by benzimidazole sites
Humidity requirement	Strongly hydration-dependent	Can operate under low or no external humidification
Typical operating temperature	Low-temperature PEMFC range, typically below 100 °C	HT PEMFC range, typically 120–200 °C
Conductivity control variable	Water uptake, RH, ionic-domain connectivity	ADL, acid-network connectivity, activation energy, polymer confinement
Main advantage	Very high conductivity under fully hydrated conditions and mature technological platform	Good conductivity under anhydrous high-temperature conditions; improved CO tolerance; F-free alternative
Main limitation	Conductivity drops sharply at low RH; water management is critical	High ADL may induce plasticization, acid migration/loss, and mechanical weakening
Dominant design challenge	Maintaining hydration while controlling swelling and mass transport	Balancing conductivity, acid retention, and mechanical stability
Structure property focus	Ionic-channel morphology and water management	Coupled transport–plasticization–retention framework

**Table 2:** Descriptor-based mapping of synthetic strategies in PA–PBI membranes. The table links each design approach to key descriptors, highlighting the associated trade-offs and their roles in balancing proton transport, mechanical stability, and durability.

Strategy	Descriptor	Effect
**Crosslinking**	ADL/acid retention	Controls free acid and limits excessive uptake
	Activation energy (E_a_)	Slight increase or near-neutral effect
	Mechanical properties	Increases stiffness and reduces swelling
	Main trade-off	Excessive crosslink density reduces segmental mobility
	Design role	Structural stabilization
**Backbone modification** (e.g., *fluorination*, *para-ordering*)	ADL/acid retention	Tunes polymer–acid interactions
	Activation energy (E_a_)	Can reduce E_a_ by improving proton transport pathways
	Mechanical properties	Increases packing efficiency and backbone cohesion
	Main trade-off	Greater synthetic complexity
	Design role	Structural control
**Functional group engineering** (e.g., *nitrogen-rich sites*)	ADL/acid retention	Enhances acid binding and retention
	Activation energy (E_a_)	Reduces E_a_ by facilitating proton transfer
	Mechanical properties	Moderate effect, depending on functionality and loading
	Main trade-off	Balancing acid stabilization with acid mobility
	Design role	Transport enhancement
**Topology engineering** (e.g., *PAFs*, *MOFs*)	ADL/acid distribution	Increases acid retention and improves spatial distribution
	Activation energy (E_a_)	Can reduce E_a_ by introducing additional transport pathways
	Mechanical properties	Increases stiffness and dimensional stability
	Main trade-off	Dispersion and compatibility challenges
	Design role	Coupled transport–stability optimization
**Free volume expansion**	ADL/acid uptake	Increases acid uptake and carrier density
	Activation energy (E_a_)	Can reduce E_a_ through enhanced local mobility
	Mechanical properties	Reduces modulus and increases swelling tendency
	Main trade-off	Higher risk of plasticization and degradation
	Design role	Conductivity enhancement
**Composite/** **hybrid systems**	ADL/acid retention	Tunable depending on filler chemistry and morphology
	Activation energy (E_a_)	Tunable
	Mechanical properties	Tunable
	Main trade-off	Interface stability and phase compatibility
	Design role	Multi-objective optimization

**Table 3:** List of abbreviations used in this review.

Abbreviation	Full Term	Description
ADL	Acid doping level	Moles of H_3_PO_4_ per polymer repeat unit (mol·RU^−1^); governs proton carrier density and network formation
E_a_	Activation energy	Energy barrier for proton transfer within hydrogen-bond networks
σ	Proton conductivity	Ionic conductivity of the membrane (S·cm^−1^)
dV/dt	Voltage decay rate	Rate of voltage loss during operation (μV·h^−1^); indicator of electrochemical durability
P_max	Peak power density	Maximum power output of the fuel cell (mW·cm^−2^)
HT-PEMFC	High-temperature proton exchange membrane fuel cell	Fuel cell operating at 120–200 °C under low or no external humidification
PEMFC	Proton exchange membrane fuel cell	General class of fuel cells employing polymer electrolytes
PFSA	Perfluorosulfonic acid	Class of fluorinated polymer membranes (e.g., Nafion)
PBI	Polybenzimidazole	Aromatic polymer backbone used in high-temperature membranes
PA–PBI	Phosphoric acid-doped polybenzimidazole	Acid-doped polymer membrane system enabling anhydrous proton transport
H_3_PO_4_	Phosphoric acid	Proton-conducting species forming hydrogen-bond networks
H_2_PO_4_^−^	Dihydrogen phosphate ion	Intermediate species participating in proton transport pathways
MEA	Membrane electrode assembly	Integrated membrane–electrode unit in fuel cell devices
RH	Relative humidity	Measure of water content in hydrated membrane systems
MW	Molecular weight	Polymer chain size influencing entanglement and mechanical properties
OCV	Open circuit voltage	Cell voltage measured under zero current conditions

**Table 5:** Mechanical and dimensional stability metrics (plasticization envelope).

Source	Membrane/Architecture	ADL (mol/RU or wt%)	Tensile Strength RT (MPa)	Tensile Strength at Elevated T (MPa, T)	Swelling/Dimensional Change (%)	Notes
[18]	High-MW PBI-78 kDa/10.8 PA	10.8 (mol/RU)	30.3	7.3 (@130 °C)	–	High mechanical robustness at high ADL
[29]	o-PBI neat	7.06 (mol/RU)	75	–	–	Reference for cavity-engineered variants
[29]	o-PBI-Zn cavity	9.54 (mol/RU)	45	–	–	Strength decreases with cavity/free-volume increase
[29]	o-PBI-Co cavity	9.76 (mol/RU)	35	–	–	Further strength reduction vs. Zn
[32]	PBI/imi-HPA composite (doped)	290.4% uptake	14–18	–	Swelling up to 180% (reported)	High conductivity but high swelling; moderate strength
[32]	QPAES-10%OA-POSS (benchmark)	11.6	8.5	2.4 (@110 °C)	–	Crosslink + filler synergy; not PBI

**Table 6:** Acid retention and durability metrics (electrochemical aging).

Source File	Membrane	ADL	Retention Metric	Test Duration (h)	Test T (°C)	Current Density/Protocol	Voltage Decay (μV/h)	OCV (V)	Notes
[30]	AmPBI-PIL (CTDTr) crosslinked, N+ site engineered	–	83% PA retention	240	160	static furnace (0% RH)	–	–	Retention at 160 °C without humidification
[32]	PBI/imi-HPA composite	290.4% uptake	Acid loss ~49–54% (steam exposure)	–	–	steam exposure	–	–	Retention limitation despite high σ
[18]	High-MW PBI-78 kDa/10.8 PA	10.8	–	–	160	300 mA/cm^2^	1.5	0.942	Best quantified durability among extracted set
[18]	PBI-54 kDa/11.2 PA	11.2	–	–	160	300 mA/cm^2^	110.1	0.923	High decay despite similar ADL
[18]	PBI-37 kDa/11.5 PA	11.5	–	<15	160	300 mA/cm^2^	–	0.865	Rapid failure; low OCV

**Table 7:** Single-cell performance benchmarking (HT-PEMFC; report conditions explicitly).

Source	Membrane/Architecture	σ (S/cm) (T, Condition)	Pmax (mW/cm^2^)	T (°C)	Ox	Catalyst Loading (mg Pt/cm^2^)	Notes
[18]	High-MW PBI-78 kDa/10.8 PA	0.14 (160 °C, anhydrous)	295	160	air	0.6	OCV 0.942 V; decay 1.5 μV/h @300 mA/cm^2^
[18]	PBI-54 kDa/11.2 PA	–	300	160	air	0.6	OCV 0.923 V; decay 110.1 μV/h
[30]	AmPBI-PIL (CTDTr) crosslinked, N+ site engineered	0.1849 (180 °C, anhydrous)	550.9	160	O_2_	~2 (anode)/~2 (cathode)	MEA 25 cm^2^; membrane thickness 67 μm; peak power @160 °C
[32]	PBI/imi-HPA composite	0.1666 (200 °C, anhydrous)	454	160	O_2_	–	Acid loss ~49–54% under steam exposure
[32]	QPAES-10%OA-POSS (benchmark)	0.0974 (180 °C, anhydrous)	461	200	O_2_	–	Non-PBI benchmark; crosslink+filler synergy
[31]	Crosslinked PBI systems (review range)	≤0.25 (160–180 °C)	400–600 (range)	160–180	var.	var.	Benchmark ranges; populate with specific exemplars during full extraction
[32]	Ph-PBI/imi-HPA-3–15% composite	0.1666 (200 °C, anhydrous)	454	160	O_2_	0.6	Thickness 80 μm; ADLs 290.4%; H_2_ 0.3 L/min, O_2_ 0.15 L/min; atm pressure.

## Data Availability

No new data were created or analyzed in this study. Data sharing is not applicable to this article.
